# Protein Structure Insights into the Bilayer Interactions of the Saposin-Like Domain of *Solanum tuberosum* Aspartic Protease

**DOI:** 10.1038/s41598-017-16734-2

**Published:** 2017-12-05

**Authors:** Brian C. Bryksa, Rickey Y. Yada

**Affiliations:** 1 Ontario Agricultural College, University of Guelph, N1G 2W1 Guelph, Ontario Canada; 2 Faculty of Land and Food Systems, University of British Columbia, Vancouver, V6T 1Z4 British Columbia Canada

## Abstract

Many plant aspartic proteases contain a saposin-like domain whose principal functions are intracellular sorting and host defence. Its structure is characterised by helical segments cross-linked by three highly conserved cystines. The present study on the saposin-like domain of *Solanum tuberosum* aspartic protease revealed that acidification from inactive to active conditions causes dimerisation and a strand-to-helix secondary structure transition independent of bilayer interaction. Bilayer fusion was shown to occur under reducing conditions yielding a faster shift to larger vesicle sizes relative to native conditions, implying that a lower level structural motif might be bilayer-active. Characterisation of peptide sequences based on the domain’s secondary structural regions showed helix-3 to be active (~4% of the full domain’s activity), and mutation of its sole positively charged residue resulted in loss of activity and disordering of structure. Also, the peptides’ respective circular dichroism spectra suggested that native folding within the full domain is dependent on surrounding structure. Overall, the present study reveals that the aspartic protease saposin-like domain active structure is an open saposin fold dimer whose formation is pH-dependent, and that a bilayer-active motif shared among non-saposin membrane-active proteins including certain plant defence proteins is nested within an overall structure essential for native functionality.

## Introduction

Unlike their non-plant counterparts, many plant aspartic proteases contain a ~100-residue insert, often referred to as the *plant-specific insert* (PSI), within the C-terminal portion of the protease. Upon post-translational processing and activation, PSIs are either released (heterodimeric plant aspartic proteases) or retained (monomeric plant aspartic proteases)^1–4^; in the latter scenario the PSI and protease moieties exist as structurally distinct features of a shared overall tertiary structure^3,5^ which function independently^5–9^. The PSI is non-proteolytic and classified structurally as a saposin-like protein^3,7,10–12^. Since the N- and C-terminal portions occur in the opposite order relative to other saposin-like proteins, PSI is also called *swaposin* ^12^. Members of the saposin-like protein superfamily have diverse biological functions^10,13–16^ all involving lipid interactions^4,10,17^. Mechanisms by which membrane bilayer-saposin-like protein contacts occur have been proposed spanning a variety of modes^18–21^, yet there is limited information regarding PSIs relative to more-studied members of the superfamily such as helminth amoebapores, mammalian saposins A–D, surfactant protein B, NK-lysin, and granulysin.

PSIs display bilayer perturbation^4,7,22^ and membrane permeabilising^23,24^ activities, and their roles *in vivo* include endosomal sorting^1,3,11,12^ and plant pathogen resistance^25^. Biochemical insights into the antimicrobial etiology of *Solanum tuberosum* aspartic protease (StAP) PSI^22,26^ revealed its interaction with, and permeabilisation of, microbial plasma membranes^23^. With respect to intracellular protein trafficking, it has been postulated that the PSI may be responsible for bringing aspartic protease precursors into contact with membranes or membrane-bound receptor proteins during Golgi-mediated intracellular transport to vacuoles^27^. Gradual acidification is present from the endoplasmic reticulum to the lytic vacuole^28^. As part of vacuolar trafficking, processing by vacuolar processing enzymes takes place during importation of proteins either in the vacuole, or en route in pre-vacuoles or multi-vesicular bodies, a process that appears to be linked to acidification^29^. It was subsequently shown that two aspartic proteases are the major vacuolar processing enzymes in potato tubers, and that the pre-vacuole and/or multi-vesicular bodies are the site(s) of processing^30^. Furthermore, it has been established that aspartic proteases are involved in plant senescence^27,31^, e.g., sweet potato aspartic protease plays a role in ethephon-mediated leaf senescence^32^. Distinct from the central vacuole, small vacuoles having intense proteolytic activity develop during *Arabidopsis* and soybean leaf senescence which have differing tonoplast composition and are comparatively more acidic^33^. Thus, characterising PSI pH-dependence and structural features modulating its bilayer interactions is important for the broader understanding of plant development/senescence and post-harvest physiology, and plant immunity in addition to rational design approaches for developing novel plant biocontrol agents and therapeutic treatments.

**Figure 1:**
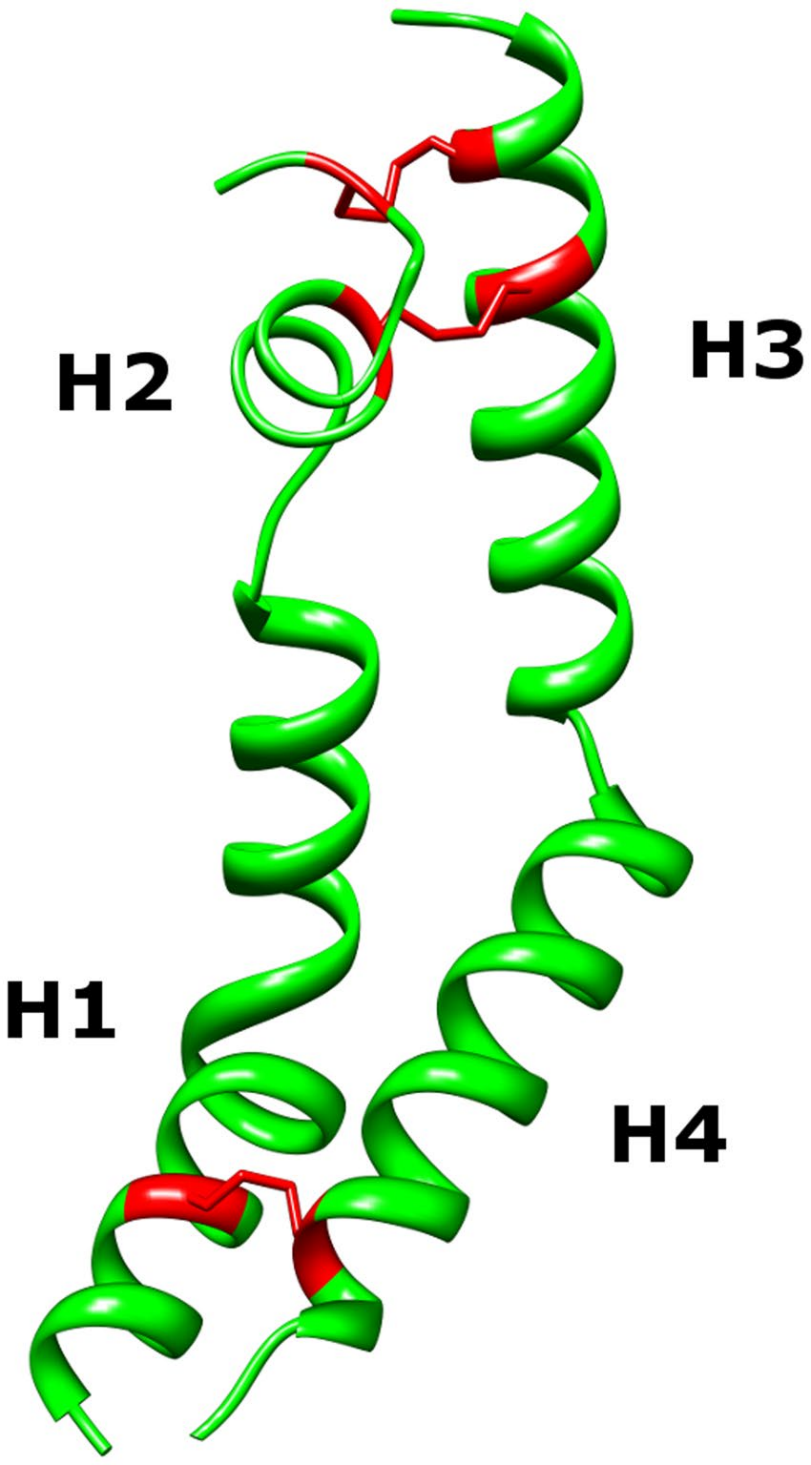
Crystal structure of StAP PSI (3RFI). The StAP PSI crystal structure was divided into structural regions based on helix content, identified as H1, H2, H3, and H4. The sequence bridging H2 and H3 was not resolved in 3RFI and is denoted as peptide “X”. The peptide sequences, and aromatic and ionisable residues are detailed in the Methods section under the heading PSI Structure Component Peptides. The positions of the three disulfide bonds (bottom to top; C6-C99, C31-C71, and C37-C68) are shown in red.

To date, the most detailed knowledge regarding PSI structure and function has been reported on StAP PSI^7,24,26,34,35^. Although it acts exclusively on anionic bilayers, StAP PSI interacts with both charged and uncharged phospholipids^24^, and causes disruption of phospholipid bilayers^7,22,24^ as do the PSIs of cardosin A^4,34^, phytepsin, and *Arabidopsis thaliana* aspartic protease^34^. StAP PSI secondary structure has been shown to be pH-dependent while independent of its disulfide bonds^7^. Its crystal structure (3RFI) revealed a dimer of extended V-shaped monomers, the less commonly observed “open” saposin fold^7^. The PSI monomer unit can be subdivided into five distinct regions (see Fig. 1); four helical regions analogous to non-plant saposin structures, and an unresolved mid-sequence region unique to plants whose structural properties remain unknown. Self-association has been shown to be critical for PSI-bilayer activity^24^, and a subsequent molecular dynamics study indicated that PSI dimerisation may be energetically driven by pH-dependent protein folding processes^35^. Thus, a better understanding of the PSI mode of action requires delineation of its quaternary structure status in solution across different pH conditions. Furthermore, the relationship between PSI secondary structure pH-dependence and bilayer activity is poorly understood. To gain insight into the structural basis for PSI-bilayer activity, the pH- and lipid bilayer-dependencies of StAP PSI as well as its tertiary structural component regions were investigated. Among the findings discussed below are the first confirmation of the structure of the active form of PSI and the structural basis for the pH-dependence of its activity, and the only report to date on the presence of a bilayer active motif normally found in certain non-saposin membrane-active proteins within a saposin-like domain.

## Results

### StAP PSI Quaternary Structure in Solution

**Figure 2:**
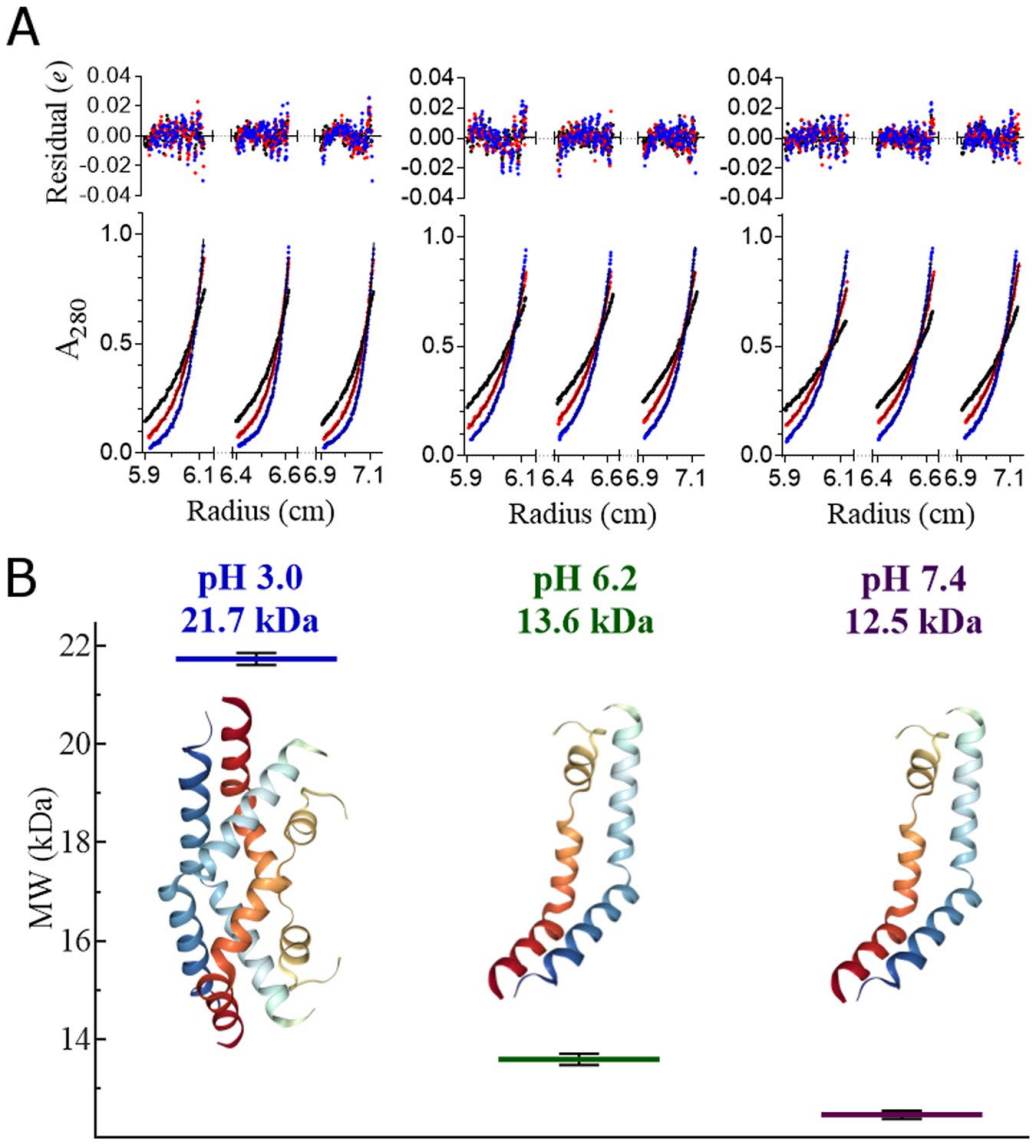
Sedimentation equilibrium analytical centrifugation analyses at pH 3.0, 6.2, and 7.4. (**A**) Absorbance measurements at 280 nm collected in 0.002 cm radial steps and averaged over 10 readings; measurements were determined in triplicate. (**B**) Summary of the calculated masses from matching tests (mean +/− one standard deviation, n = 6). The respective buffers used were 27 mM sodium phosphate pH 3.0, 20 mM sodium phosphate pH 6.2, and 10 mM sodium phosphate pH 7.4, all containing 140 mM NaCl.

**Figure 3:**
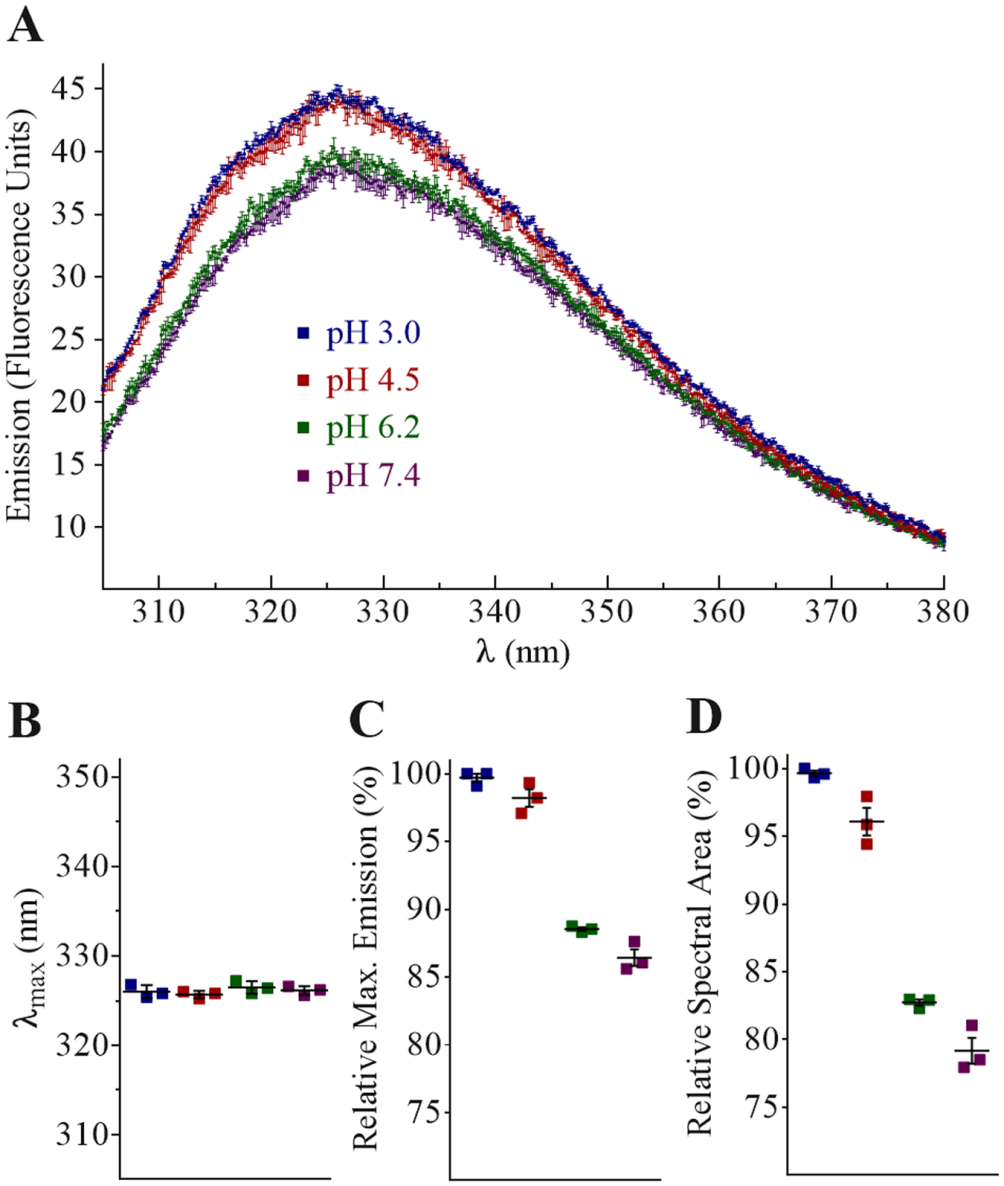
Intrinsic Trp fluorescence emission. (**A**) Solution intrinsic Trp fluorescence emission of StAP PSI at pH 3.0, 4.5, 6.2, and 7.4. (**B**) λ_max_ at the indicated pH values were not significantly different comparing all respective pairings (P > 0.05). (**C**) Relative maximum fluorescence emission was not significantly different (P > 0.05) between pH 3.0 and 4.5, nor pH 6.2 and 7.4 (P > 0.05), whereas each of the former pair were significantly different (P ≤ 0.05) from the latter pair, respectively. (**D**) Relative areas under the emission spectra (305–380 nm) were significantly different (P ≤ 0.05) for all data set pairings (mean +/− one standard deviation, n = 3).

Although StAP PSI (3RFI) crystallised as a dimer^7^, its tertiary/quaternary solution structure in active and inactive conditions remains unknown. With a predicted pI 4.6, PSI solubility near optimal pH for bilayer activity^4^ was insufficient for analytical centrifugation. As a compromise, PSI was analysed at pH 3.0, 6.2, and 7.4, and was determined to have apparent masses of 21.7 and 13.6 and 12.5 kDa, respectively (Fig. 2). These results indicated that recombinant PSI (11,771 Da) exists exclusively as a dimer in the active pH range, and as a monomer closer to neutrality. To determine the PSI monomer-dimer status at pH 4.5, intrinsic tryptophan fluorescence emission was used (Fig. 3A) which was similar to that at pH 3.0 (dimer) and distinct from the monomer form observed at pH 6.2 and 7.4. Although the pH 3.0 dimer had stronger fluorescence emission, optimal emission wavelength remained unchanged (Fig. 3B).

### Secondary Structure Dependence on pH and Disulfide Bonds

**Figure 4:**
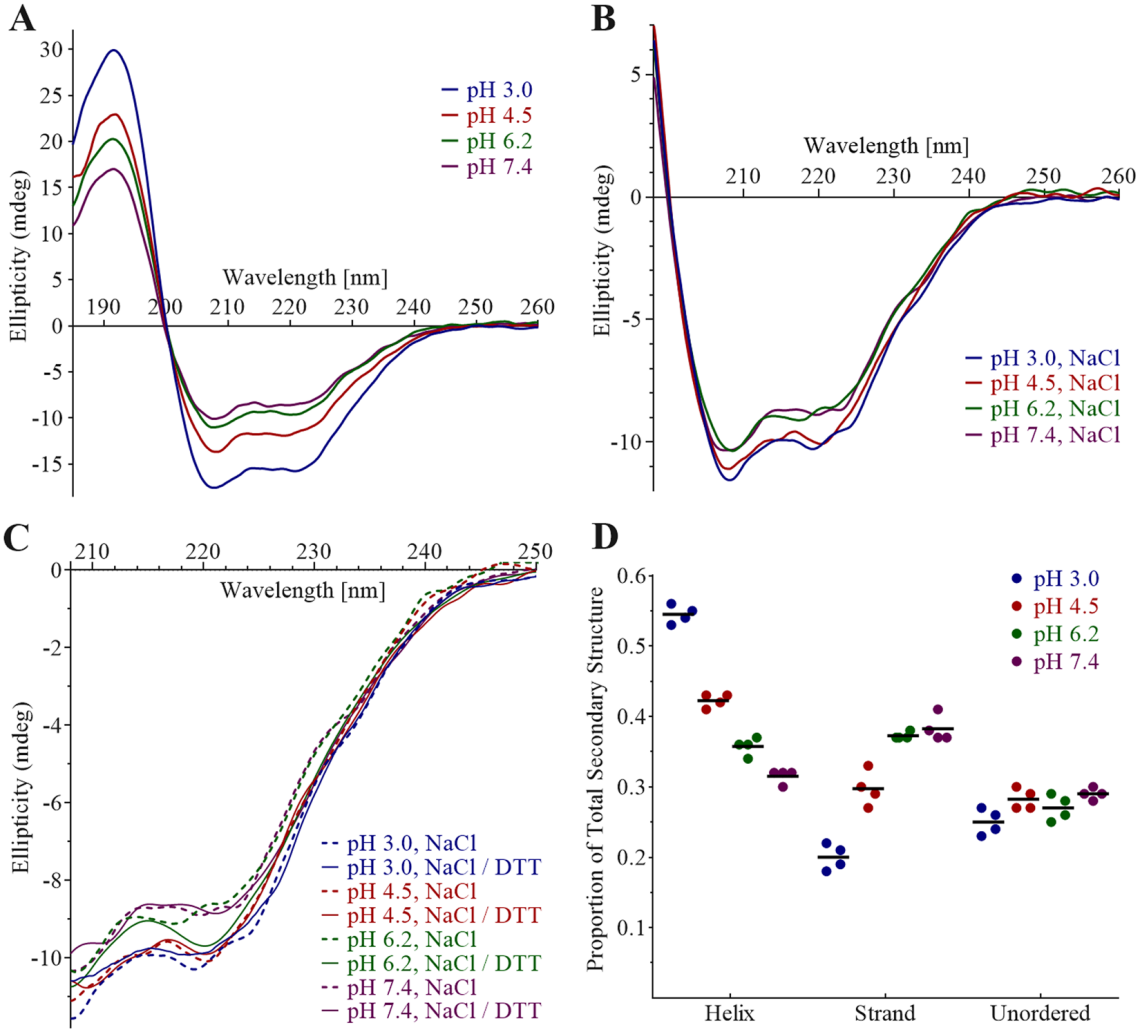
pH-dependence of PSI secondary structure. Far-UV CD spectra of 100 µg/mL (8.5 µM) StAP PSI were measured at pH 3.0 (blue), 4.5 (red), 6.2 (green), and 7.4 (purple) collected in (**A**) buffer; (**B**) buffer containing 140 mM NaCl; and (**C**) buffer containing 140 mM NaCl and 10 mM DTT, overlaid with spectra from (**B**). The respective buffers used were 27 mM sodium phosphate pH 3.0, 50 mM sodium acetate pH 4.5, 20 mM sodium phosphate pH 6.2, and 10 mM sodium phosphate pH 7.4. Secondary structure contents calculated for PSI in buffer are summarised in (**D**).

PSI secondary structure contents as a function of pH were compared using far-UV circular dichroism (CD) in iso-ionic buffers under non-saline, saline, and reducing saline conditions (Fig. 4A–C, respectively). Trends of increasing helix content with decreasing pH (P ≤ 0.05 for each paired comparison), and an accompanying increase in strand/turn content with increasing pH (pH 3.0 < 4.5 < 6.2, P ≤ 0.05 for each paired comparison; pH 6.2 and 7.4 not significantly different, P > 0.05), were observed. Unordered secondary structure content did not appear to follow any clear pattern based on pH (Fig. 4D). Dominant features particularly evident in the pH 3.0 and 4.5 spectra included a large positive peak centred below 195 nm and two narrow large negative peaks at 222 nm and 208 nm, indicators for high helix content^36^. Increasing pH resulted in reduced intensities of these peaks and a broadening of the local minima in the 222–208 nm region. Spectra collected under reducing and non-reducing saline conditions were suitable only for qualitative comparisons due to a high level of noise caused by DTT and NaCl at wavelengths below 208 nm and 196 nm, respectively, as well as increasing UV absorbance by DTT below 225 nm. Differences in low pH saline spectra relative to their non-saline counterparts also suggested that secondary structure sensitivity across the pH range was somewhat mitigated by saline conditions. Although the presence of saline conditions did not appear to change the pattern of higher helical character at lower pH values, saline spectra occurred in two discrete secondary structure states (i.e., indistinguishable spectra pairings at pH 3.0 and 4.5, and pH 6.2 and 7.4, respectively). Although signal:noise below 208 nm was limiting, the PSI CD spectra under reducing conditions seemed to indicate similar overall secondary structures (Fig. 4C).

### pH-Dependence of PSI-induced Phospholipid Bilayer Disruption

**Figure 5:**
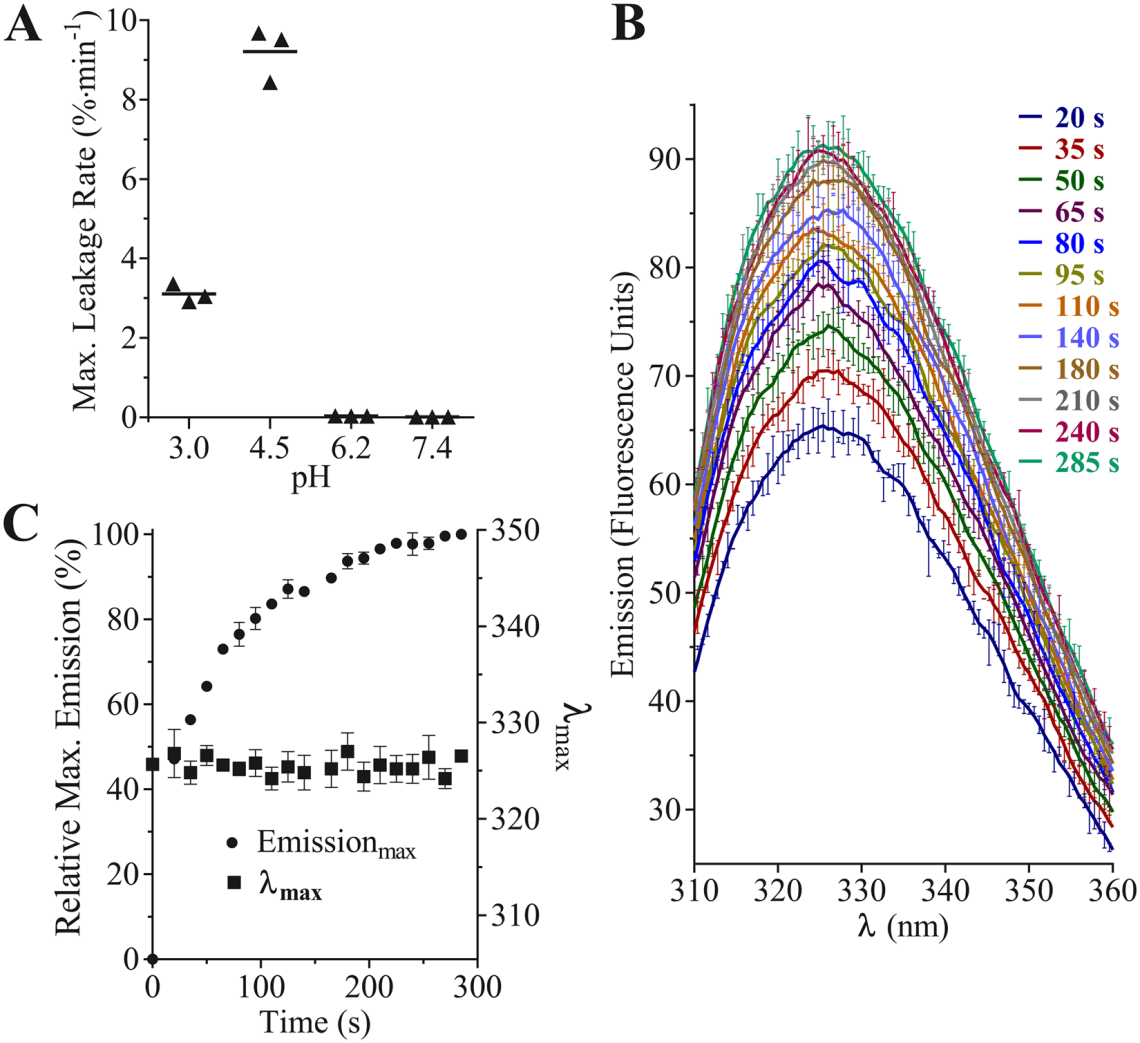
**(A**) pH-dependence of PSI-induced bilayer vesicle leakage at 25 °C in 27 mM sodium phosphate pH 3.0, 50 mM sodium acetate pH 4.5, 20 mM sodium phosphate pH 6.2, or 10 mM sodium phosphate pH 7.4, all containing 140 mM NaCl. Maximum leakage rates are expressed as a function of fluorescence increase relative to Triton X-100-lysed LUV controls in the respective pH conditions. Disruption was detected at pH 3.0 and 4.5, but not at pH 6.2 and 7.4. Activity assessments shown used 0.5 µM PSI at active pH values and 10 µM PSI at inactive values. The higher concentrations used for the latter was to confirm that no leakage could be detected at pH 6.2 and 7.4. (**B**) Intrinsic Trp fluorescence spectra of 10 µM StAP PSI with 1:1:1 POPC:POPE:POPS bilayer vesicles (100 µM total phospholipid). (**C**) Summary of maximum fluorescence and λ_max_, indicating no shift in λ_max_ despite the increased emission (mean +/− one standard deviation, n = 3).

Bilayer disruption pH-dependence was assessed using fluorophore-loaded large unilamellar vesicles (LUVs; 100 µM total phospholipid) and 0.5–10 µM PSI (Fig. 5A). Leakage was monitored until cessation of fluorescence increase. PSI caused vesicle leakage at pH 3.0 and 4.5, with the latter having a 3-fold higher maximum leakage rate (significantly different, *P* ≤ *0.05*). After approximately 15 min, PSI-induced leakage consistently plateaued at approximately 25% of maximum fluorescence. Leakage was not detected at pH 6.2 and 7.4 (not significantly different, *P* > *0.05*).

### PSI Intrinsic Tryptophan Fluorescence Emission and Secondary Structure in the Presence of Anionic Bilayer

**Figure 6:**
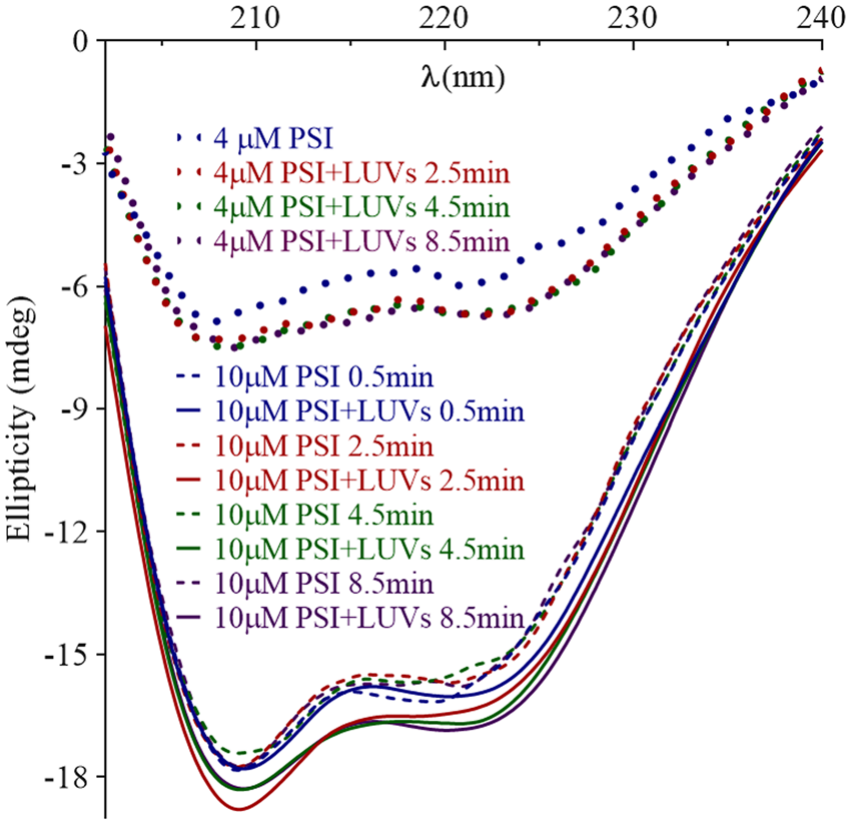
PSI secondary structure upon incubation with bilayer. Far-UV CD spectra were measured over 30 min for PSI incubated with 1:1:1 POPC:POPE:POPS bilayer vesicles (100 µM total phospholipid) in 50 mM sodium acetate/140 mM NaCl buffer pH 4.5 at 22 °C. Overlapping scans are omitted for clarity.

PSI Trp fluorescence and secondary structure upon encountering bilayer vesicles (100 µM total phospholipid) were monitored over time in buffered saline at optimal pH (Fig. 5B,C). Maximum fluorescence emission followed a single phase non-linear association (R^2^ = 0.99), increasing steadily for approximately 1 min followed by a deceleration period that was near static after ~5 min (Fig. 5C). λ_max_ did not change (i.e., no significant departure from zero slope; P > 0.05). With respect to PSI secondary structure, continuous monitoring resulted in excessive far-UV exposure such that coagulation of the LUV mixture into a gel-like state occurred. Therefore, CD spectra were collected in a discontinuous manner and at a relatively rapid scan rate to minimise exposure (Fig. 6). Phospholipid-containing controls and samples were verified by dynamic light scattering (DLS) to confirm respective expected vesicle sizes after CD assays. Overlapping spectra beyond 8.5 min were omitted for clarity. The principal change in CD spectra over time was a modest but consistent increase in ellipticity for PSI-bilayer mixtures compared to PSI in solution. At 222 nm and 208 nm, ellipticity increased by approximately 26% and 16% for 10 µM PSI, and 13% and 4% for 4 µM PSI, respectively. Spectral changes were essentially complete in less than 2.5 min.

### Characterisation of PSI-Induced Bilayer Effects: Cryo-Transmission Electron Microscopy

**Figure 7:**
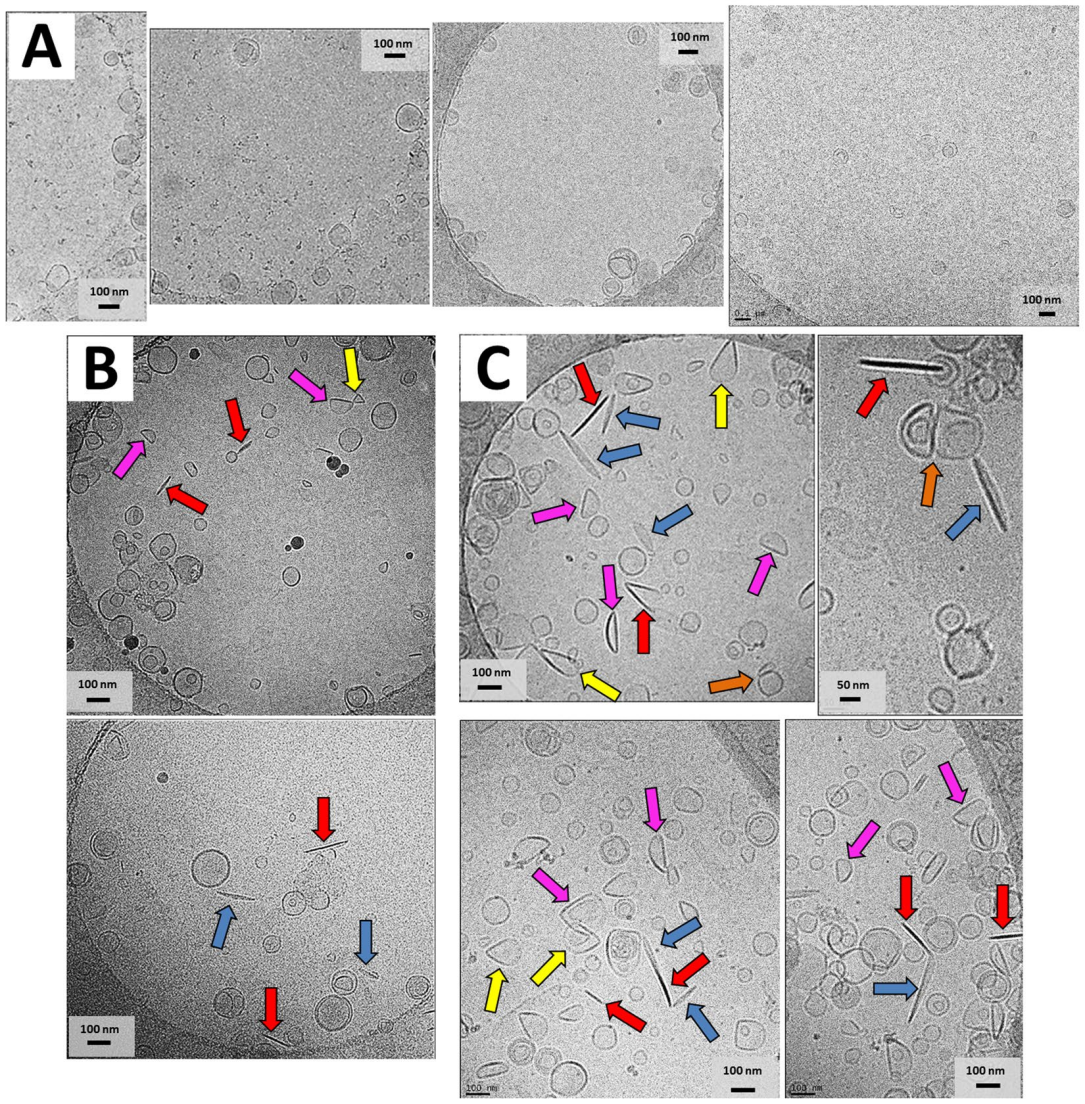
Cryo-transmission electron microscopy. Shown are images representative of the observed phenomena for 1:1 POPE:POPS bilayer vesicles in 50 mM sodium acetate/140 mM NaCl buffer pH 4.5, incubated for 15 min at 22 °C; (**A**) untreated; (**B**) 500 µM phospholipid/10 µM PSI; and (**C**) 1000 µM phospholipid/16 µM PSI. Different observed morphologies are identified by arrows as follows: Oblong structures that appear to be partially-collapsed vesicles (blue arrows); individual liposomes with a flattened or straight edge (pink arrows); vesicles interfacing at respective flat edges (orange arrows); wedge-type vesicles having two flat edges meeting at an end (yellow arrows); and narrow elongated rod-like structures approximately 100–200 nm in length (red arrows).

**Figure 8:**
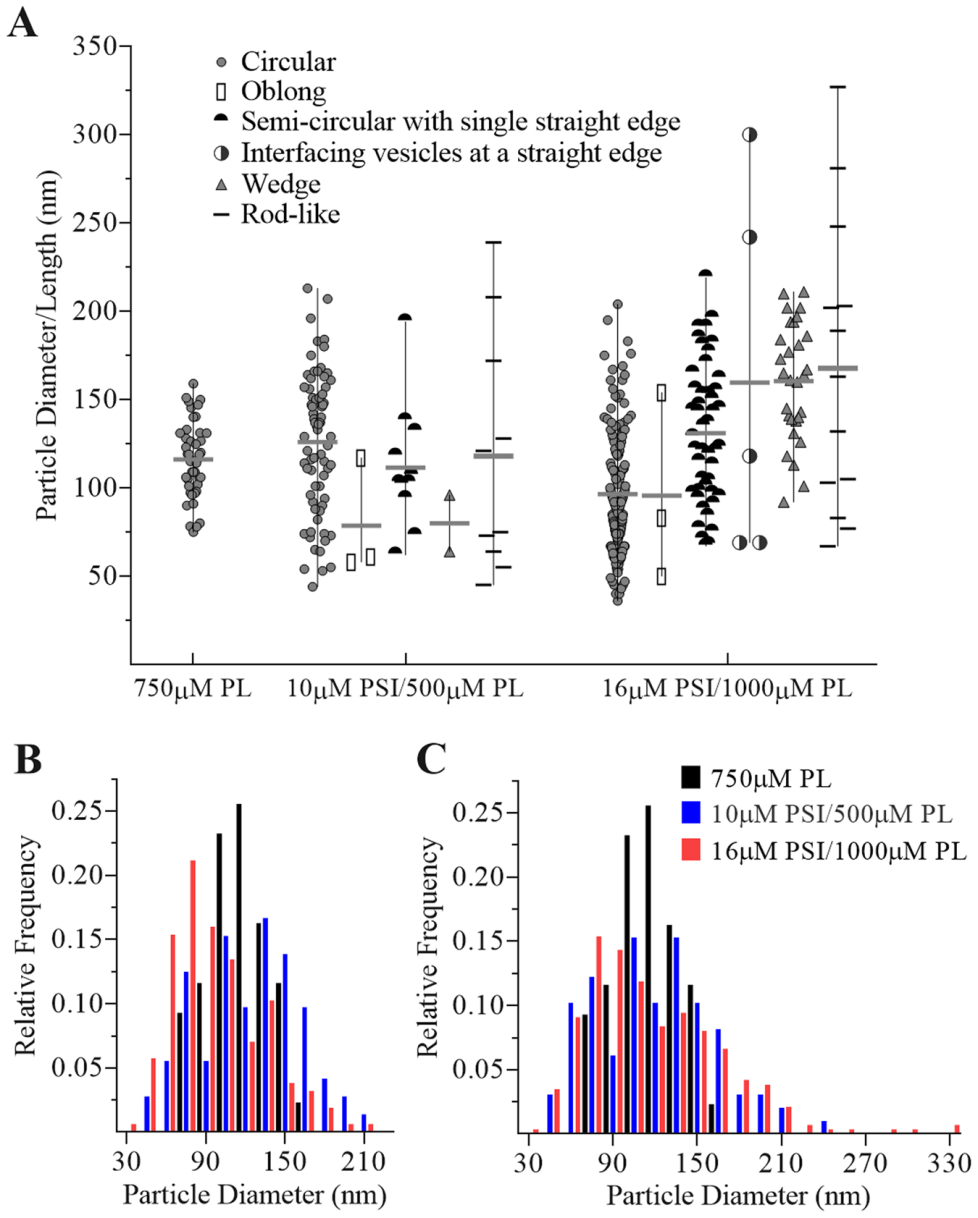
Summary of cryo-TEM image morphological phenomena. (**A**) Enumeration and comparison by size of vesicle morphologies as identified in the legend; (**B**) histogram comparison of circular vesicle size distributions; and (**C**) histogram comparison of all vesicle morphologies’ size distributions. Note that results are derived from images not limited to the selected examples in Fig. 8, and exclude vesicles in contact with the sample grid.

Imaging was used to characterise liposome morphology upon PSI treatment (see Fig. 7). Images were acquired for controls (vesicles alone) and 15 min PSI-vesicle incubations at two concentrations of PSI and phospholipid (10 µM PSI/500 µM phospholipid and 16 µM PSI/1000 µM phospholipid) at 22 °C. Vesicles substantially contacting the sample support grid were excluded from analyses due to typical artefactual vesicle morphologies^37^. Control vesicle diameters agreed with DLS measurements of approximately 125–140 nm. Dramatic changes were noted for PSI-treated vesicles, and were categorised into 5 distinct morphologies: (i) narrow oblong structures that appear to be partially-collapsed vesicles (blue arrows); (ii) individual semi-circular liposomes with a flattened or straight edge (pink arrows); (iii) vesicles interfacing at respective flat edges (orange arrows); (iv) wedge-type vesicles having two flat edges intersecting at their ends (yellow arrows); and (v) narrow elongated rod-like structures (red arrows). The rod-like structures in particular appeared to show well-defined bilayer by way of a thin low-contrast layer surrounding a high density thin centre, suggesting a collapsed liposome. Vesicle morphologies from suitable (9 control and 8 test) cryo-transmission electron microscopy (TEM) images were enumerated and analysed (Fig. 8). Histograms of vesicle diameter distributions for circular vesicles as well as for overall vesicle populations were summarised, and analyses of variance indicated that the respective vesicle diameter distributions were significantly different (P ≤ 0.05) for both comparisons, respectively (Fig. 8B,C). Comparing the circular vesicle size distributions (Fig. 8B), both 10 µM PSI/500 µM phospholipid and 16 µM PSI/1000 µM phospholipid tests were significantly larger (P ≤ 0.05) than control, respectively, as well as each other. The appearance of larger circular vesicles confirmed bilayer fusion.

### PSI Bilayer Fusion Activity: Dynamic Light Scattering

**Figure 9:**
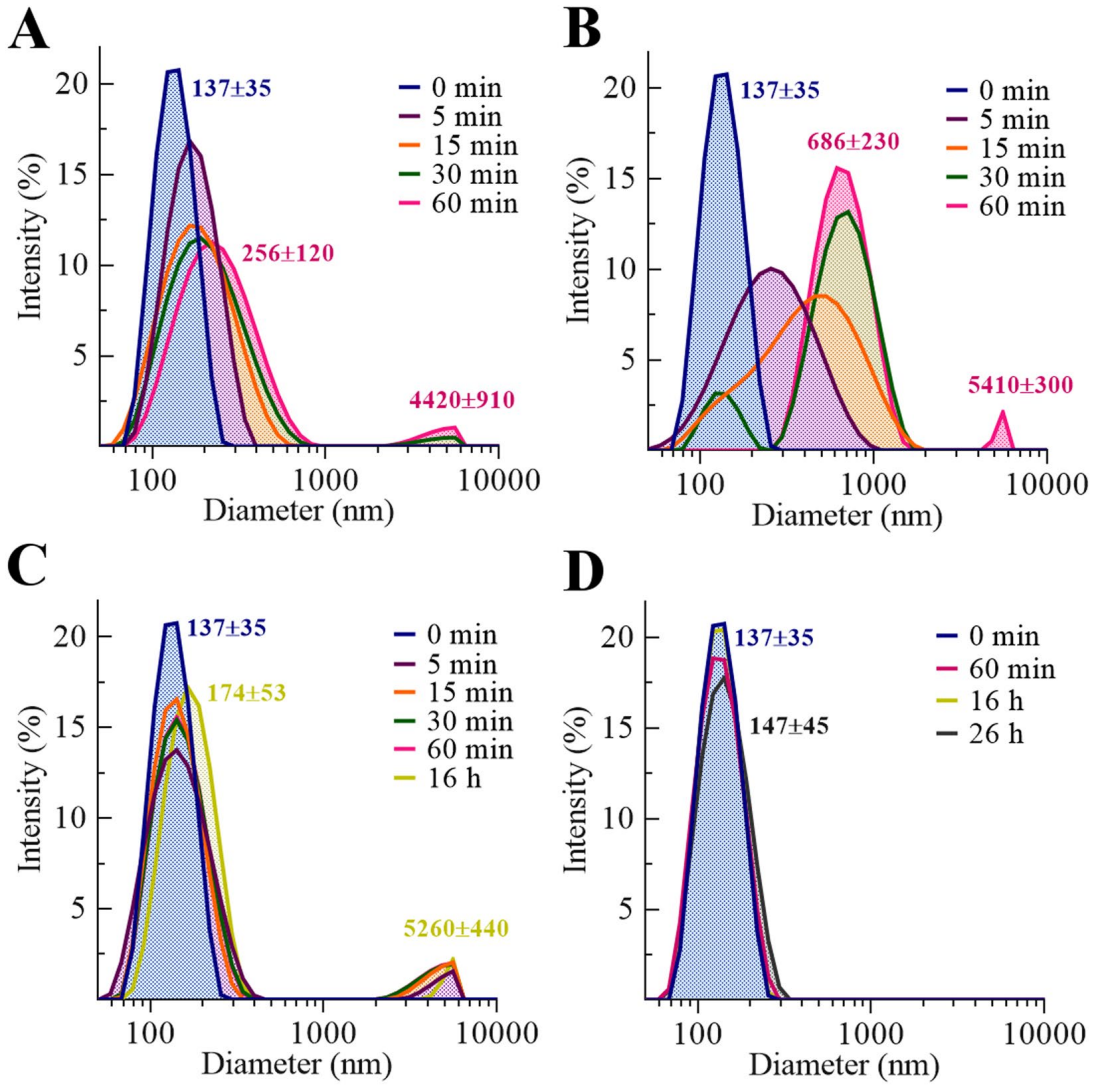
Vesicle size monitored by DLS. (**A**,**B**) PSI-treated vesicle size was monitored over time using 1:1 POPE:POPS LUVs (100 µM phospholipid) incubated in 50 mM sodium acetate/140 mM NaCl buffer pH 4.5 with 10 µM PSI under (**A**) non-reducing or (**B**) reducing (10 mM DTT) conditions. (**C**,**D**) H3-induced vesicle fusion was also assayed using (**C)** 10 µM H3 and (**D**) 10 µM K83Q H3 in the same manner as in (**B**).

DLS was used to measure vesicle size over time as a means of monitoring fusogenic activity, as has been used previously^7,38,39^. Assays were carried out at room temperature for up to 26 h, or until polydispersity became excessive or aggregation was detected. LUV size was monitored in both non-reducing and reducing buffered saline (Fig. 9A,B, respectively). PSI caused peak broadening within 5 min, and by 30 min, a population of relatively large particles (4000 ± 1000 nm) constituted ~2% of the vesicle population with the remainder in a size range that averaged almost double the original size (220 ± 100 nm). Fusion assays for PSI in reducing conditions also initially shifted the vesicle population to higher average diameter, however, a broader and larger diameter population formed more quickly compared to native PSI. In the absence of its disulfide bonds, PSI appeared to produce intermediate size products relative to native conditions, and shifted the entire vesicle population as opposed to the more modest and gradual broadening of the diameter range for native PSI.

### PSI Component Peptide Bilayer Disruption

**Figure 10:**
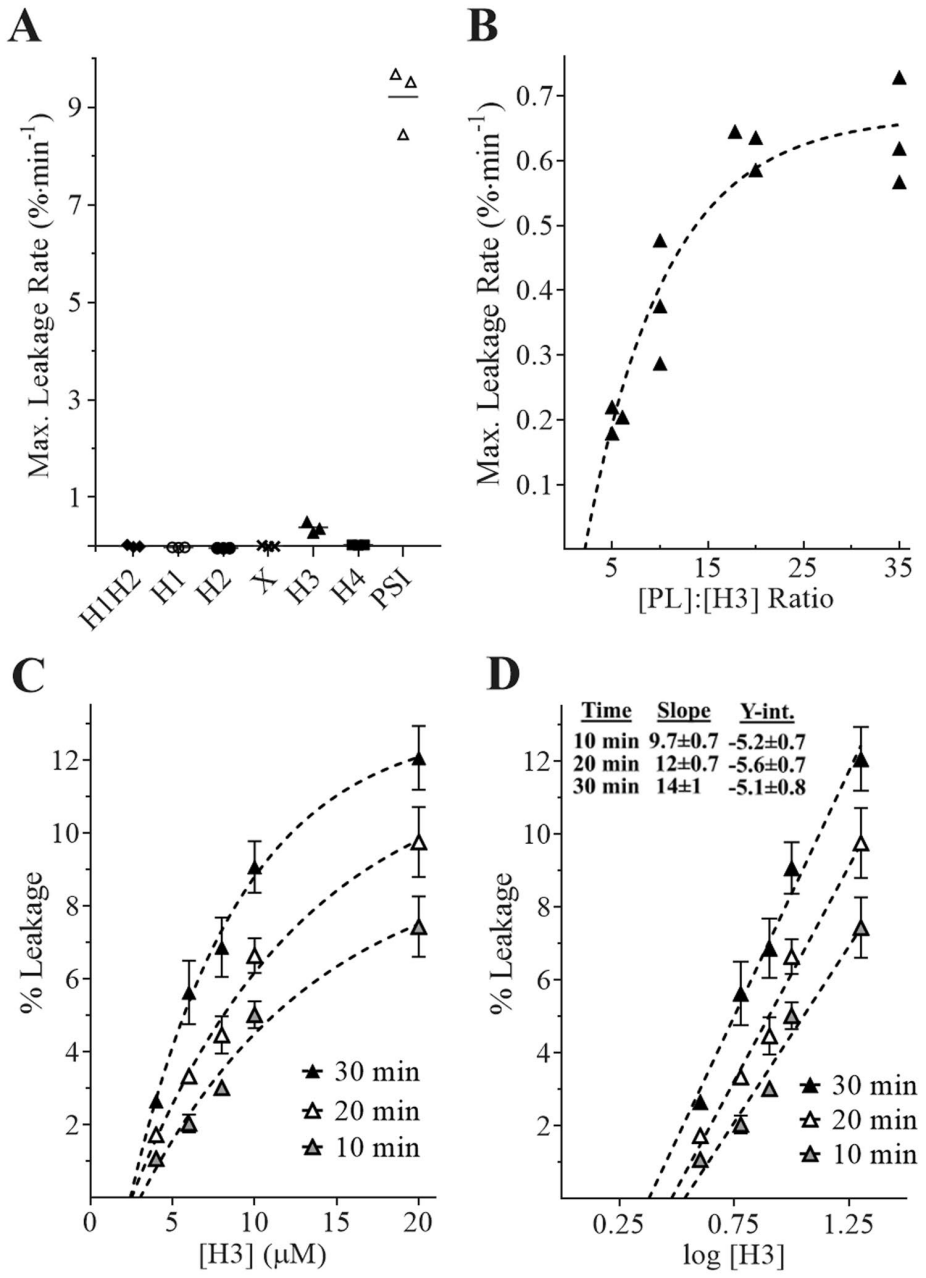
Leakage assays for peptide-treated 1:1 POPE:POPS bilayer vesicles in 50 mM sodium acetate/140 mM NaCl buffer pH 4.5 at 25 °C. (**A**) Peptide screening assays for vesicle leakage activity used 40 µM H1H2, H2, X, and H5; and 10 µM H1 (low solubility of H1 prevented screening above 10 µM). The bilayer disruption activity of PSI is shown for comparison. (**B**) Leakage rate-dependence on [phospholipid]:[H3] ratio; and (**C**,**D**) Extent of leakage induced by 4–20 µM H3 (mean +/− one standard deviation, n = 3). Leakage was non-linearly proportional to H3 concentration, and semi log curves were significantly different (P ≤ 0.05).

Peptides corresponding to the PSI secondary structure subdivisions outlined in Fig. 1 were screened for bilayer disruption activity under reducing conditions (Fig. 10A), revealing that H3 induced vesicle leakage (~4% of the full PSI’s activity) while the other peptides showed no indication of bilayer disruption at concentrations up to 40 µM (except H1 whose low solubility prevented testing beyond 10 µM). Combining peptides in native or reducing conditions had no additive/synergistic effect (data not shown). Like PSI, fluorescence emission for H3 leakage assays plateaued at approximately 25% relative to positive control, however, rates were slower than full-length PSI (Fig. 5A). H3 maximum leakage rate was confirmed to be non-linearly dependent on [phospholipid]:[H3] ratio (Fig. 10B). Insight into the mode of H3-induced bilayer disruption was sought by relating peptide concentration to the extent of leakage at three time points (Fig. 10C,D). The semi-log analyses (Fig. 10D) revealed distinct (P ≤ 0.05) linear curves having increasing slopes (P ≤ 0.05) and similar intercepts (P > 0.05) (see inset table in Fig. 10D). Solving for the x-intercepts, there appeared to be a critical peptide concentration (average 2.9 ± 0.5 µM) necessary for leakage/disruption activity. H3 charge mutants E64Q, E72Q, and K83Q were also designed with the latter resulting in complete loss of activity whereas low solubility prevented characterisation of the two Glu mutants.

### H3 Intrinsic Tryptophan Fluorescence Emission in the Presence of Anionic Bilayer

**Figure 11:**
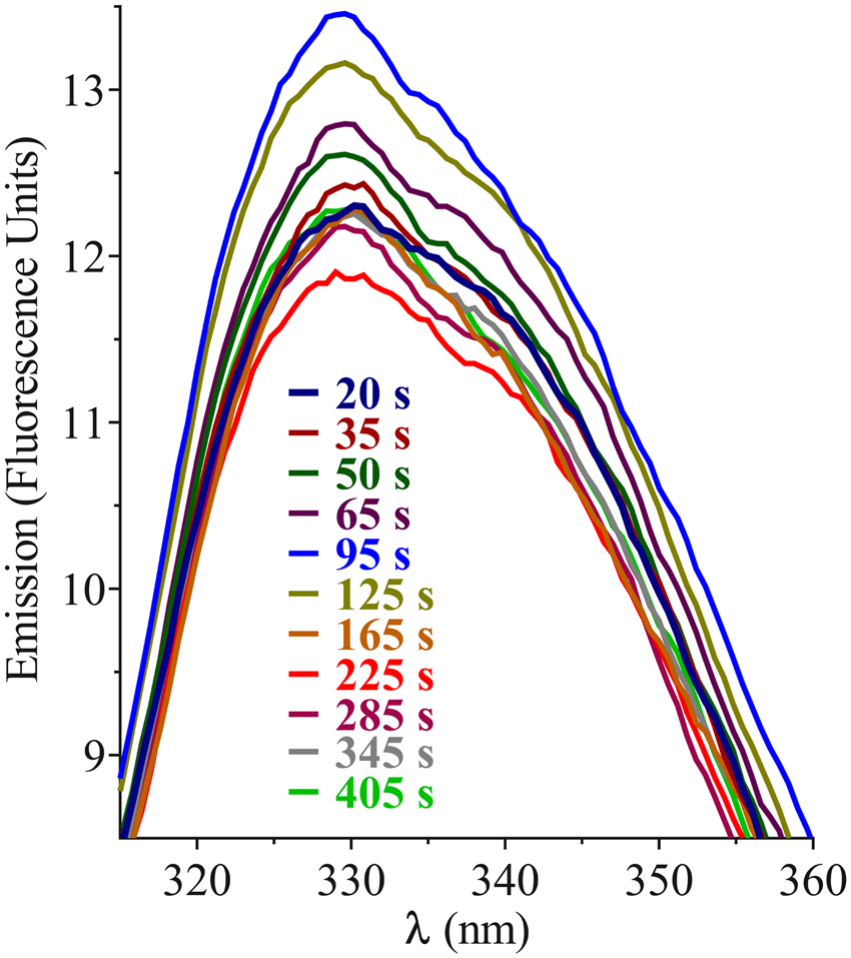
Intrinsic Trp fluorescence emission spectra upon incubation of 10 µM peptide H3 with 1:1:1 POPC:POPE:POPS bilayer vesicles (100 µM phospholipid) in 50 mM sodium acetate/140 mM NaCl buffer pH 4.5 at 25 °C. Each spectrum is the average of 3 scans, and differences in λ_max_ and Emission_max_ were not significantly different (P > 0.05). Overlapping scans are omitted for clarity.

Trp emission for the sole active component peptide was measured upon incubation with bilayer vesicles (Fig. 11). Like PSI, λ_max_ did not shift, and fluorescence emission initially increased; however, unlike PSI, emission subsequently trended down after 95 s, overshooting the start scan and eventually equilibrating to near the start point. Although changes between consecutive scans seemed to indicate a coherent progression, scans were all within error of one another, leading to the conclusion that H3 bilayer penetration was unlikely.

### Secondary Structure of PSI Component Peptides

**Figure 12:**
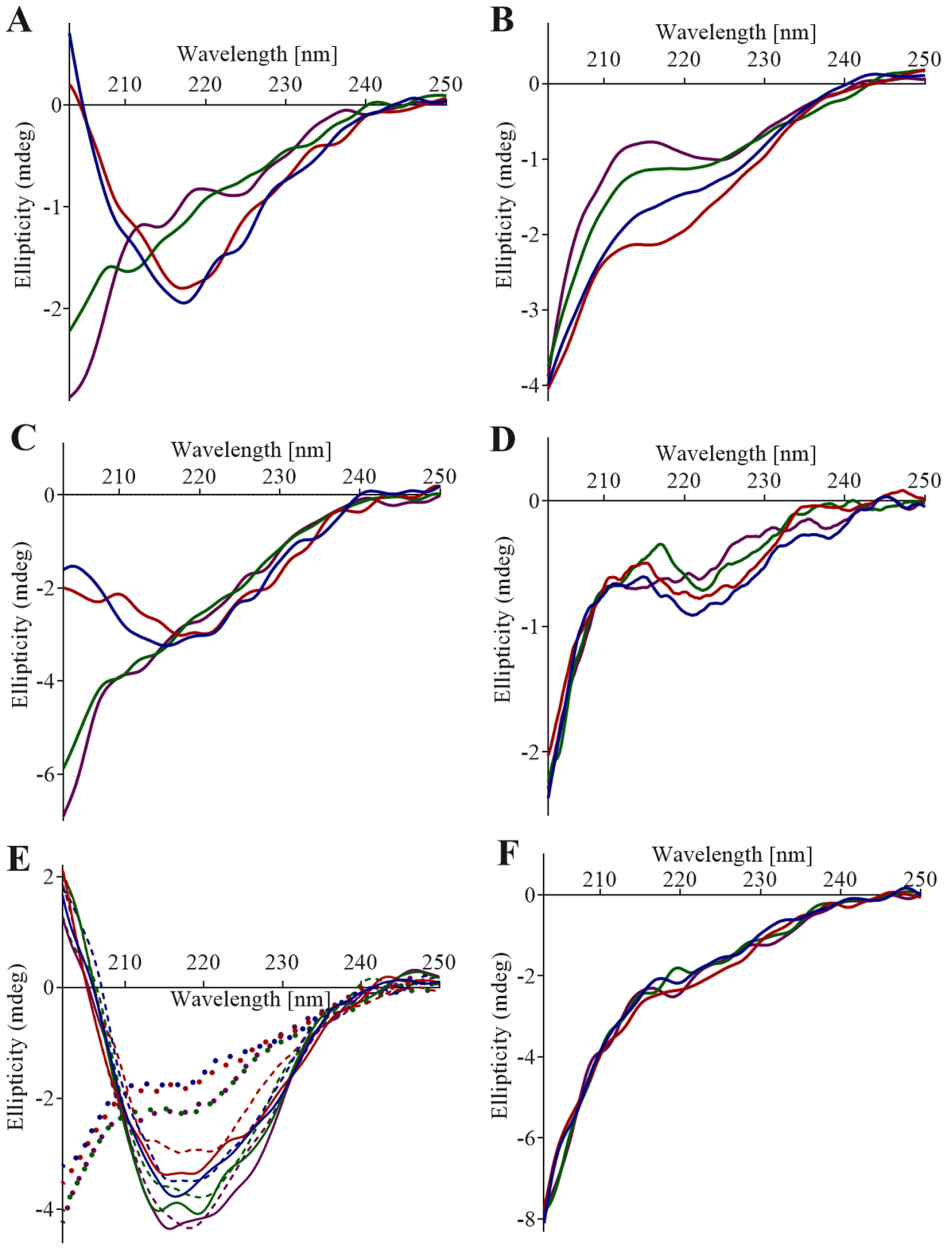
Far-UV CD spectra of peptides in buffered saline (140 mM NaCl) containing 10 mM DTT at pH 3.0 (blue), 4.5 (red), 6.2 (green), and 7.4 (purple); (**A**) H1; (**B**) H2; (**C**) H1H2; (**D**) X; (**E**) H3 (solid lines), H3 incubated with 1:1:1 POPC:POPE:POPS LUVs (100 µM phospholipid; broken lines), K83Q H3 (circles); and (**F**) H4. The buffers used were 27 mM sodium phosphate pH 3.0, 50 mM sodium acetate pH 4.5, 20 mM sodium phosphate pH 6.2, and 10 mM sodium phosphate pH 7.4.

Peptides’ CD spectra were collected under reducing conditions (Fig. 12) to avoid interaction via free Cys residues, precluding quantification of secondary structures due to far-UV absorbance below 215 nm. Qualitatively^36,40^, the spectra for H3 (Fig. 12E) had characteristics suggestive of high strand/turn content (ellipticity peak near 218 nm and loss of ellipticity at wavelengths >208 nm) mixed with helix (substantial ellipticity in the 225–240 nm region). The K83Q H3 mutation resulted in a drastic disordering of H3 secondary structure (Fig. 12E). The overall shapes of the H3 spectra did not appear to be substantially affected by phospholipid bilayer nor changes in pH. By contrast, the H1 and H1H2 spectra (Fig. 12A,C, respectively) indicated distinct secondary structure pH-dependence such that predominant strand/turn (ellipticity peak near 218 nm and decreasing ellipticity >208 nm) was indicated in the active pH range, whereas extensive disordering occurred at inactive pH values (strongest ellipticity below 208 nm). Also, higher helix content for H1H2 over H1 was apparent (broader curve and relatively stronger ellipticity below 218 nm) at active pH values. H2, X, and H4 (Fig. 12B,D,F, respectively) were predominantly disordered (ellipticity peak below 208 nm) mixed with helix and strand/turn (net negative ellipticity in the 210–240 nm range).

### Peptide-Induced Vesicle Fusion

As was the case for leakage, only peptide H3 induced bilayer vesicle fusion (Fig. 9C,D). Vesicle size profiles differed somewhat from those for PSI (Fig. 9A,B). H3 caused relatively slower broadening of the original vesicle population, but immediate appearance of very large particles (4% abundance by 5 min). Also, as observed for leakage activity, the K83Q mutation abolished apparent fusion activity, showing no discernible change in vesicle size through 26 h.

## Discussion

Insights into PSI-induced effects on anionic bilayers were gained through DLS and cryo-TEM analyses. These included broadening of vesicle size range and transformation of spherical liposomes to several distinct product structures. Vesicle size distributions from cryo-TEM images generally agreed with those measured by DLS in that vesicle size range broadened to both smaller and larger diameters. Furthermore, the appearance of enlarged individual spheroid vesicles in cryo-TEM images, taken together with the DLS and TEM size distributions’ similarities, provided direct evidence of PSI-mediated vesicle fusion. The vesicle morphologies detailed in Figs 8 and 9, when considered in the context of TEM image interpretation reported previously^41,42^, may represent an apparent progression of different stages of vesicle collapse wherein partial flattening of liposomes leads to eventual elongated electron-dense structures. Interestingly, the semi-circular single-edge morphology is a known phenomenon regarding fusogenic protein-vesicle initial interactions^43^, and has also been reported for melittin-induced vesicle effects^44^. The observed flattening of vesicle bilayers was consistent with vesicle morphologies that result from phospholipid bilayers subjected to phase transitioning^45^, suggesting that PSI altered bilayer liquid-gel transition. This cryo-TEM-based finding supports previous colorimetry-based measurements of PSI lowering of lipid transition temperature^24^. Additionally, the mechanism of the observed PSI-induced bilayer remodelling to flat surfaces (e.g., Fig. 7C, red arrows) would be expected to involve PSI oligomerisation since the PSI extended open structure^7^ measures only ~6–9 nm in its longest dimension^7,35^. PSI self-association upon mixing with vesicle bilayers, detected by infrared spectroscopy, has been reported at similar concentrations and protein:lipid ratios^24^, albeit using different phospholipid mixtures. Further evidence of oligomerisation was provided by the time-dependent increase in Trp fluorescence upon mixing PSI and bilayer (Fig. 5B,C) which equilibrated to a distinct end-point. The fact that this process was time-dependent concurred with the previously-reported dependence of bilayer disruption on PSI-bilayer interaction time, which is suggested to allow for protein lateral diffusion during oligomerisation for pore formation as per the Barrel-Stave model^24^. Such an arrangement could also account for incomplete leakage noted in the present study since pore formers would be situated in a fraction of vesicles thereby causing leakage in just some targets while others remain intact (i.e., all-or-none leakage)^46^.

With respect to protein-protein contacts, dimer formation has been implicated in saposin-membrane interactions^47–50^, and StAP PSI has been reported to be monomeric in hydrophilic conditions while forming oligomers in hydrophobic environments^22^. Saposins A and C form acidic pH-dependent dimers and trimers, respectively^51^, and saposin B exists as a dimer regardless of pH^47^. StAP PSI, like the latter, has bilayer activity at low pH, and a high degree of similarity exists between the StAP PSI^7^ and saposin B^47^ structures which adopt the open saposin fold, unusual among saposin-like proteins. Sedimentation equilibrium (Fig. 2) and Trp fluorescence (Fig. 3) experiments on PSI in lipid-free solution revealed a dimer at pH 3.0 and 4.5, and monomer near neutral pH with only a small proportion of dimer at pH 6.2. Furthermore, an increase in helix structure at pH 3.0 and 4.5 appeared to accompany dimerisation for lipid-free PSI (Fig. 4), and increased ellipticity near 222 nm and 208 nm suggested a further increase in helix content upon interaction with bilayer (Fig. 6) over a similar amount of time as Trp emission increase (Fig. 5B,C). One possible explanation for the lack of blue shift in Trp emission scans was an *internal Stark effect* ^52^ which can be manifested if a positively charged residue becomes positioned near the Trp benzene ring, or a negative charge near the pyrrole ring, resulting in a λ_max_ red-shift via a process outside of the intended experimental design. This situation may be present in PSI as there are several charged residues proximate to Trp.

The overlap of the above CD and Trp emission data with the pH dependence of PSI bilayer activity led to the conclusion that, in addition to protein structure changes upon bilayer interactions, changes in PSI structure yielding dimerisation also must occur prior to encountering bilayer. Thus, protein oligomerisation in PSI-bilayer interactions, discussed above, must begin from the “jackknife” open dimer structure. We postulate that such an arrangement could serve to provide a protein fold that is soluble in aqueous intracellular environments while containing a large hydrophobic surface (interfaced with the paired monomer) poised to make intermolecular hydrophobic contacts without a need for more extensive unfolding from a compact monomeric classical saposin fold. Also noteworthy was that the pH range for the monomer-dimer transition was consistent with that which spans the pathway from Golgi bodies to the vacuole^53,54^. Perhaps this relates to modulation or specificity of targeting within the endosomal system considering that aspartic proteases have been confirmed to be vacuolar processing enzymes in pre-vacuole/multi-vesicular bodies^30^, in addition to their presence within vacuoles.

Although the CD spectra for native and reducing conditions indicated no role for disulfide bonds in overall secondary structure at active pH values (no apparent change in spectra), and DLS profiles (Fig. 9A,B) indicated that disulfide bonds were non-essential for vesicle fusion, their reduction did result in non-native fusion (i.e., vesicles shifted to discrete populations having greater average size compared to non-reducing conditions). We hypothesised that the disulfide bond-free PSI structure may have allowed for increased accessibility to bilayer-interacting structural features, and therefore interaction may be dictated, at least in part, by a motif (or motifs) within the PSI structure. Upon screening PSI structural component peptides for activity, the finding that H3 has bilayer disruption and fusion activities was unexpected as it contains just a single positively charged residue unlike the other helical regions within PSI. Three charge-eliminating mutants were subsequently tested (i.e., E64Q, E72Q and K83Q) revealing that the sole Lys is essential for activity while the two Glu residues each substantially impact on the solubility of the sequence. Component regions of saposin C have been studied previously^55^ with some notable differences. For saposin C, H1 and H2 (equivalent to PSI H3 and H4, respectively) did not cause bilayer fusion. Comparing sequences, PSI H3 is more hydrophobic than either of the saposin C peptides, and although the positive charge content is inferior for PSI H3, its amphipathic charge distribution together with the hydrophobic *AVVW* middle patch may explain the different fusogenic abilities for the respective regions of these two otherwise similar saposin-like proteins:

$$\begin{array}{c}{\rm{StAP}}\,{\rm{PSI}}\,{\rm{H3}}\,-\,\underline{{\rm{E}}}{\rm{APL}}{\bf{C}}{\rm{TA}}{\bf{C}}\underline{{\rm{E}}}{\rm{M}}{AVVW}{\rm{MQNQL}}\underline{{\bf{K}}}{\rm{Q}}\\ {\rm{Saposin}}\,{\rm{C}}\,{\rm{H1}}\,-\,{\rm{YC}}\underline{{\rm{E}}}{\rm{VC}}\underline{{\rm{E}}}{\rm{FLV}}\underline{{\bf{K}}}{\rm{EVT}}\underline{{\bf{K}}}{\rm{LID}}\\ {\rm{Saposin}}\,{\rm{C}}\,{\rm{H2}}\,-\,\underline{{\rm{E}}{\bf{K}}}{\rm{EIL}}\underline{{\rm{D}}}{\rm{AF}}\underline{{\rm{D}}{\bf{K}}}{\rm{MCS}}\underline{{\bf{K}}}{\rm{LP}}\underline{{\bf{K}}}\end{array}$$

The pH-induced increase in helix content observed for PSI in the present study would be expected to be localised within some portion of the overall structure since the PSI N- and C-terminal halves occur in the opposite order relative to saposins (i.e., H1-H2-H3-H4; H3-H4-H2-H1) yet share similar pH dependencies, bilayer charge requirement for activity (e.g., saposins C and D)^23,47,49,51,56–62^, and structures (e.g., saposins B and C)^7,47,48^. CD scans for the component peptides appeared to identify the N-terminal helix as the source of pH-sensitivity since ordered structure was gained at pH 3.0 and 4.5 from largely disordered neutral pH secondary structures for both H1 and H1H2 (Fig. 12). The structures of H1 and H2 were clearly distinct from H1H2, and furthermore, peptides’ helix contents in general did not appear to account for that of the overall PSI structure. Thus, secondary structures within PSI would seem to be at least partially dependent on their presence within the greater tertiary structure.

While PSI underwent a strand/turn-to-helix transition upon acidification (Fig. 4D) and concomitant dimerisation (Fig. 2), H3, the only bilayer-active peptide, seemingly had no substantial helix content nor drastic pH-induced rearrangement (Fig. 12). Furthermore, the increased Trp hydrophobicity in PSI upon bilayer interaction, stable after several minutes (Fig. 5B,C), contrasted with H3 which appeared to experience short-lived changes only to return to its initial status and remaining static thereafter (Fig. 11). This indicated a non-penetrating mechanism of action for H3, and together with the CD results, indicated that PSI and H3 may have different mechanisms of action. From analysis of H3 bilayer disruption (Fig. 10), the apparent requirement for attaining a critical peptide concentration to initiate leakage suggested a cooperative mechanism of action. Furthermore, H3 appeared to have characteristics common to other types of membrane-active peptides including magainins^63–65^ and amyloid peptides^66,67^. Similarities to magainins include: H3 yielded abnormally slow rates of vesicle leakage^63,68^; such activity increased dramatically with peptide concentration once the concentration threshold was met; leakage slowed with time for all peptide concentrations^63,69^; and leakage ceased prior to attaining completion^68^. H3 may operate by a carpet-like mechanism^69^ wherein a cooperative bilayer-perturbing process results in leakage of vesicle contents, functionality which subsequently dissipates due to peptide equilibration across the bilayer^63,69^. Amyloid-β peptide-induced vesicle leakage also proceeds only to partial leakage^70,71^. H3 appeared to have predominantly strand/turn secondary structure (Fig. 12E), and perhaps more importantly, certain amyloid peptides, proposed to employ the carpet mechanism in some situations^72,73^, produce similar distortions in anionic bilayer vesicle morphology (i.e., the appearance of flat surfaces and elongated vesicles)^70,72,74^. The implications of these similarities remain to be elucidated in future investigations.

Inspection of the sequence features of H3 include certain noteworthy elements as follows:

$$\underline{{\rm{E}}}{\rm{APLCTAC}}\underline{{\rm{E}}}{\rm{M}}{AVVW}{\rm{M}}{\bf{QNQL}}\underline{{\bf{K}}}{\bf{Q}}{\rm{.}}$$

Despite its short 21-residue length, H3 is zwitterionic (charged residues underlined) as well as amphiphilic with a central hydrophobic patch (italics). Additionally, the sole positive charge (bold underlined), shown to be essential for activity in the present study, is surrounded by multiple Gln or Asn residues at the C-terminus. Interestingly, this overall arrangement is shared with certain other plant defence-related proteins including the flocculating cationic peptide from *Moringa oleifera* seed shown to damage bacterial membranes via a membrane fusion mechanism^75^, and a 2S albumin from *Ricinus communis* which causes leakage of bacterial cells postulated to result from magainin-like pore formation^76^ (see Table 1). A motif query (avoiding redundancy) of the RCSB Protein Data Bank for the H3 C-terminal 6-residue motif [N/Q]-[N/Q]-[N/Q]-[A/L/I/V]-[K/R]-[N/Q] yielded a total of just 28 proteins, all lipid-interacting or surface-active (see Supplementary Table S1 for a listing of these matches). It thus appears that the above sequence motif, shared among distantly-related organisms from viruses, bacteria, and animals, may serve a plant defence-related functionality in PSI as well as flocculant peptides and 2S albumins.

**Table 1:** Examples of antimicrobial/membrane-interacting proteins that contain the [N/Q]-[N/Q]-[A/L/I/V]-[R/K]-[N/Q] sequence motif (bold underlined italic). Sequences similar to the motif (bold italic) and hydrophobic patches (italic) are also indicated.

Protein	Sequence	Ref.
StAP PSI H3	EAPLCTACEM*AVVW*M***QNQLKQ***	^90^
Mabinlin I	EPLCRRQFQQHQHLRACQRYIRRRAQRGGLVDEQRGPALRLCC***NQLRQ***VNKPCVCPVLRQAAH***QQLYQ***GQIEGPR***QVRQ***LFRAARNLPNICK*IPAV*GRCQFTRW	^91^
Mabinlin II	QPRRPALRQCC***NQLRQ***VDRPCVCPVLRQAA***QQVLQRQ***IIQGP***QQLRR***LFDAARNLPNICNIPNIGACPFRAW	^81,92^
Flocculent-active protein MO2.1 and MO2.2	QGPGRQPDFQRCG***QQLRN***ISPPQRCPSLRQAVQLTHQQQGQVGP***QQVRQ***MYRVASNIPST	^93^
*Moringa oleifera* CBP_3_	CPAIQRCC***QQLRN***IQPPCRCCQ	^94^
Sesame 2S albumin (*Sesamum indicum*)	MAKK*LALAAVLLVAMVALA*SATTYTTTVTTTAIDDEANQQS***QQCRQ***QLQGRQFRSCQRYLSQGRSPYGGEEDEVLEMSTGNQQSEQSLRDCC***QQLRN***VDERCRCEAIRQAVRQQQQEGGYQEGQSQQVYQRARDLPRRCNMRPQQCQFR*VIFV*	^95^
* β-lactoglobulin fragment 1−8	*LIV*T***Q***TMK* This example is shown because it contains the skeleton of the motif in question: hydrophobic patch, glutamine or asparagine, single positive charge	^96^

Additionally, a protein domain structure alignment search of the RCSB PDB structure database for PSI (3RFI; using the Protein Structure Comparison Tool v.4.2.0^77^ running the FATCAT^78^ algorithm) produced high-scoring matches to diverse surface-active/membrane-interacting proteins including endosomal/vacuolar factors (endosomal sorting complex required for transport-I^79^ and vacuolar transporter chaperone 4^80^, a seed storage albumin (sweet protein mabinlin-2^81^), and calcium sensing and sodium channel protein domains (calmodulin and associated C-terminal domain^82^, and cp-aequorin^83^) (see Supplementary Fig. S1 for alignment details). The PSI structure alignments to the latter group is particularly interesting due to its utilisation of the calcium binding protein EF-hand motif (i.e., a helix-loop-helix segment)^84^, reminiscent of the previously-reported N-terminal kinked-helix within PSI^7^. Also noteworthy is that the only protein that contains the H3 structural motif among the high-scoring structural alignments is mabinlin-2, a 2S seed storage albumin that is predominantly helix and highly crosslinked (4 cystines) similar to saposin-like proteins. The best overall structure alignment match for PSI was the central region (E234–S304) of the Vps23 subunit of endosomal sorting complex required for transport-I (ESCRT-I)^79^, a ubiquitous and conserved endosomal protein critical for sorting ubiquitinated cargo into multi-vesicular bodies in yeast, mammals, and even retroviruses^85,86^. Considering the critical role of PSI for vacuolar targeting in plants, and lipid bilayer interactions more generally, it is plausible that the ESCRT-I-like tertiary structure fold is important to PSI functionality.

In conclusion, inspection of the structural elements that make up StAP PSI has offered insights into its membrane interaction requirements. The present study showed that its active form is a dimer (2 interfacing open saposin fold monomers) while it is monomeric near neutral pH where it has no activity. Dimerisation is accompanied by a concomitant helix-to-turn transition, both independent of lipid presence. Reduction of the PSI’s disulfide bonds did not cause apparent secondary structure changes, and although vesicle fusion profiles were altered a relatively high level of activity was produced by the reduced form compared to non-reducing conditions. Furthermore, nested within its primary structure, PSI contains a regional sequence that is itself bilayer-active. Oddly, this peptide on its own takes up a β-structure configuration as opposed to its usual helical character within PSI, a situation that may be related to the relatively weak bilayer disruption activity compared to full length PSI. H3 also deserves further investigation as it may represent the basis for novel membrane active protein applications (e.g., nano-particle targeting), irrespective of its apparent central role in PSI-bilayer structure-function. Although the very hydrophobic N-terminal region is shown to be intrinsically pH-sensitive, overall tertiary and/or quaternary structures of PSI are essential to the native folding of each component region regardless of pH. The singular remaining source of acidic pH-induced gain in helix in PSI appears to be the extensive hydrophobic contacts that reside within the dimer, a connection that should be addressed in future studies by a systematic approach targeting both hydrophobic regions as well as ionisable residues. More broadly, the noted structural cohesion among surface/lipid-active proteins across protein superfamilies calls for comprehensive comparative structural investigations into the potential transposability of their common functionalities.

## Methods

### Materials

A *PSI* synthetic gene optimised for expression in *E. coli* was purchased from Mr. Gene GmbH (Regensburg, Germany). Plasmid pET32b(+), *E. coli* Rosetta-gami B (DE3)pLysS, and u-MAC^TM^ 5 × 1 mL cartridges were obtained from EMD Biosciences (San Diego, CA, USA). *E. coli* TOP10F’ was from Invitrogen (San Diego, CA, USA). A GenElute^TM^ Plasmid Miniprep Kit was obtained from Sigma-Aldrich (St. Louis, MO, USA). QIAquick^®^ PCR Purification and Gel Extraction Kits were from Qiagen (Germantown, MD, USA). Restriction enzymes, T4 DNA ligase and *Pfu* DNA polymerase were obtained from Fermentas Life Sciences (Burlington, ON, Canada). Primers were synthesised by Sigma Genosys (Oakville, ON, Canada), and thrombin and dialysis tubing were purchased from Fisher Scientific (Ottawa, ON, Canada). A 1 mL MonoQ column and a 3 mL RPC column were from GE Healthcare (Piscataway, NJ, USA). Phospholipids were from Avanti Polar Lipids (Alabaster, AL, USA).

### PSI Expression and Purification

PSI was expressed and purified as described previously^7^. Briefly, PSI was sub-cloned into pET32b such that the interceding sequence between the thrombin cleavage and multiple-cloning sites was deleted, and expressed in *E. coli* Rosetta-gami B (DE3)pLysS. Cell lysate soluble fractions were subjected to cobalt affinity chromatography (u-MAC^TM^ 5 × 1 mL cartridges) followed by dialysis (3000 MWCO tubing) against 5 mM Tris-HCl buffer pH 7.4 (~1 × 10^5^-fold dilution). Thrombin was added to the dialysates at a 1:2000 mass ratio and incubated for least 12 h at ambient temperature (22 °C) followed 3 passes through cobalt affinity cartridges to remove the Trx fusion tag. Flow-through was dialyzed as above, then separated by anion exchange chromatography (1 mL MonoQ column) using a 0–500 mM NaCl gradient in 10 mM Tris-HCl pH 7.4. Eluent sample was further purified and desalted by reversed-phase chromatography (3 mL RPC column) using a 2% acetonitrile/0.065% TFA to 80% acetonitrile/0.05% TFA gradient over 30 column volumes. Sample was diluted 2-fold in water, flash-frozen in liquid nitrogen, and then lyophilised for 16–24 h. The PSI product was verified by SDS-PAGE and amino acid analysis (Advanced Protein Analysis Center, The Hospital for Sick Children, Toronto, ON, Canada). PSI stock solutions were prepared as needed by dissolving into 2 mM Tris-HCl/140 mM NaCl buffer pH 7.4. PSI concentration was determined by A_280_ and adjusted accordingly to 80 µM PSI.

### Preparation of Large Unilamellar Vesicles

LUV stocks were prepared as per^7^ with the exception that dried phospholipid mixtures were resuspended in 80 mM calcein solution (25 mM sodium acetate/140 mM NaCl buffer pH 4.5). Phospholipids used were 1-palmitoyl-2-oleoyl-sn-glycero-3-phosphocholine (POPC), 1-palmitoyl-2-oleoyl-sn-glycero-3-phosphoethanolamine (POPE) and 1-palmitoyl-2-oleoyl-sn-glycero-3-phosphoserine (POPS). Although vesicle preparations were prepared fresh, LUV stability in terms of vesicle diameter over several days is shown in Supplementary Fig. S2.

### Circular Dichroism Spectropolarimetry

CD analysis of PSI secondary structure was carried out using a Jasco J-810 spectropolarimeter (Jasco Inc., Easton, MD, USA). Samples (200 µL of 100 µg/mL PSI or 200 µg/mL peptide) were scanned over 180–260 nm at 100 nm/min, 0.5 s response, standard sensitivity, and at ambient temperature using a 1 mm pathlength quartz cell. Buffers (27 mM sodium phosphate pH 3.0, 50 mM sodium acetate pH 4.5, 20 mM sodium phosphate pH 6.2, and 10 mM sodium phosphate pH 7.4) with or without 140 mM NaCl were degassed under vacuum. Secondary structure contents were calculated using DICHROWEB^40^ with SELCON3^87^, and CDSSTR^88,89^ algorithms, and the mean residue weight (109.0 g/mol) and molecular weight of the recombinant protein (11768.7 g/mol). For CD time trial scans of PSI in the presence of phospholipid bilayer, 4 µM (47 µg/mL) or 10 µM (118 µg/mL) PSI mixed with 100 nm LUVs (100 µM total phospholipid; 1:1:1 molar ratio of POPC:POPE:POPS) were scanned at 2 min intervals over 196–260 nm and a scan rate of 200 nm/min.

### Large Unilamellar Vesicle Disruption Assays

PSI-caused perturbation of LUVs was measured by calcein leakage as detected using a Victor2 1420 Multilabel Counter (Perkin Elmer, Waltham, MA, USA) at 25 °C. 200 µL reactions were set up in 96-well microplates with varying concentrations of LUVs, 0.5–10 µM StAP PSI, or 4–40 µM peptides in either 27 mM sodium phosphate pH 3.0, 50 mM sodium acetate pH 4.5, 20 mM sodium phosphate pH 6.2, or 10 mM sodium phosphate pH 7.4 containing 140 mM NaCl. Peptide concentrations up to 40 µM were screened for disruption activity with the exception of H1 whose low solubility limited tests to 10 µM peptide. Leakage was detected using excitation at 385 nm and emission at 435 nm with 3 s shaking between readings. Leakage was monitored until increase in fluorescence emission ceased (15–120 min). Extent of leakage was calculated relative to end points (i.e. 100% release of vesicle contents) measured for LUVs incubated in 0.5% Triton X-100 at the respective pH values. Non-linear regression analyses were done using GraphPad Prism 6 (GraphPad Software Inc., La Jolla, CA, USA).

### Bilayer Fusion Assays

Ten μM PSI or peptide was incubated with 100 nm LUVs (100 μM total phospholipid; 1:1 molar ratio of POPE:POPS) at 22 °C in either 50 mM sodium acetate/140 mM NaCl pH 4.5 or 10 mM sodium phosphate/140 mM NaCl pH 7.4 (control). Mixtures were then monitored for changes in average LUV size by dynamic light scattering in a Malvern Zetasizer Nano-S (Malvern Instruments, Malvern, Worcestershire, UK) using a disposable polystyrene 1.5 mL semi-microcuvette. Three consecutive measurements of five runs (30 s per run) were averaged using the refractive index for polystyrene. Considerations for using DLS to characterise PSI/peptide-induced vesicle effects are discussed in Appendix B.

### Sedimentation Equilibrium Analytical Centrifugation

Sedimentation equilibrium studies were carried out using a Beckman Optima XL-A Analytical Ultracentrifuge (Biomolecular Interactions and Conformations Facility, University of Western Ontario, Canada). An *An*-60Ti rotor and six-channel cells with Eponcharcoal were used at 22,000 rpm, 28,000 rpm and 35,000 rpm, 20 °C. To achieve adequate detectability, 26–28 µM PSI were compared in 27 mM sodium phosphate pH 3.0, 20 mM sodium phosphate pH 6.2 or 10 mM sodium phosphate pH 7.4, each containing 140 mM NaCl. Absorbance measurements at 280 nm were collected in 0.002 cm radial steps and averaged over 10 readings. Solvent densities (ρ) were calculated using SEDNTERP software. Partial specific volume (ν) of the protein was calculated from its amino acid sequence to be 0.13337 mL/g. Data were analyzed using a single ideal species model in GraphPad Prism.

### Tryptophan Intrinsic Fluorescence Emission Spectrometry

Fluorescence emission spectra were recorded using a Shimadzu RF-540 spectrofluorophotometer (Shimadzu Corporation, Kyoto, Japan) with a 1-cm quartz three-sided ultra-micro cuvette in a water-circulating, temperature-controlled cell holder at 25 °C.  The settings used were λ_excitation_ 295 nm with 3 nm slit width and λ_emission_ scan 300–400 nm with 3 nm slit width. PSI stock solution (80 µM PSI in 2 mM Tris-HCl buffer pH 7.4/140 mM NaCl) was diluted 4-fold into 50 mM sodium acetate pH 4.5/140 mM NaCl and allowed to incubate in a water bath for 15 min at 25 °C as was done for a working stock of anionic LUVs (1000 µM total phospholipids) to pre-equilibrate. Ten µM PSI and 100 µM phospholipid (1:1:1 molar ratio of POPC:POPE:POPS as 100 nm LUVs) were mixed in 50 mM sodium acetate pH 4.5/140 mM NaCl and monitoring began at 20 s. Scans were completed at 500 nm/min allowing for a scan every 15 s.

### Cryo-Transmission Electron Microscopy

Liposome imaging was done at The Microscopy Imaging Facility, University of Guelph Advanced Analysis Centre (Guelph, ON, Canada) on an FEI Tecnai G2 F20 TEM (FEI Co., Hillsboro, OR, USA) with a bottom mount Gatan 4k CCD camera and 200 kV field emission. Samples consisted of 1000 µM or 500 µM phospholipid (as 100 nm LUV; 1:1 molar ratio of POPE:POPS) with or without 10 µM or 16 µM PSI in 50 mM sodium acetate pH 4.5/140 mM NaCl incubated for 15 min at 22 °C. Samples were loaded onto sample grids and plunge flash frozen in a dust-free moisture-controlled work space, and subsequently maintained at or below −170 °C.

### PSI Structure Component Peptides

Synthetic peptides (>95% purity) corresponding to the PSI structure regions outlined in Fig. 1 as well as H3 mutants E64Q, E72Q, and K83Q (full length PSI numbering) were purchased from GenicBio Ltd. (Shanghai, China). Peptide sequences are given below with acidic (underlined italics), basic (underlined), and aromatic (underlined bold) residues indicated along with molar absorptivities in brackets:

$$\begin{array}{l}{\rm{H}}1:\,\quad \quad \quad {\rm{I}}{\rm{V}}{\rm{S}}{\rm{M}}\mathop{E}\limits_{\_}{\rm{C}}{\rm{K}}{\rm{T}}{\rm{I}}{\rm{V}}{\rm{S}}{\rm{Q}}\mathop{{\bf{Y}}}\limits_{\_}{\rm{G}}\mathop{E}\limits_{\_}{\rm{M}}{\rm{I}}\mathop{{\bf{W}}D}\limits_{\_}{\rm{L}}{\rm{L}}{\rm{V}}{\rm{S}}{\rm{G}}\,(6990\,{{\rm{M}}}^{-1}{{\rm{c}}{\rm{m}}}^{-1})\\ {\rm{H}}2:\,\quad \quad \,\,\,\,\,{\rm{V}}\mathop{{\rm{R}}}\limits_{\_}{\rm{P}}\mathop{D}\limits_{\_}{\rm{Q}}{\rm{V}}{\rm{C}}{\rm{S}}{\rm{Q}}{\rm{A}}{\rm{G}}{\rm{L}}{\rm{C}}\mathop{{\bf{F}}}\limits_{\_}{\rm{V}}\,({\rm{n}}{\rm{o}}\,{\rm{a}}{\rm{b}}{\rm{s}}{\rm{o}}{\rm{r}}{\rm{p}}{\rm{t}}{\rm{i}}{\rm{v}}{\rm{i}}{\rm{t}}{\rm{y}})\\ {\rm{H}}1{\rm{H}}2:\quad \,\,\,\,\,{\rm{I}}{\rm{V}}{\rm{S}}{\rm{M}}\mathop{E}\limits_{\_}{\rm{C}}{\rm{K}}{\rm{T}}{\rm{I}}{\rm{V}}{\rm{S}}{\rm{Q}}\mathop{{\bf{Y}}}\limits_{\_}{\rm{G}}\mathop{E}\limits_{\_}{\rm{M}}{\rm{I}}\mathop{{\bf{W}}D}\limits_{\_}{\rm{L}}{\rm{L}}{\rm{V}}{\rm{S}}{\rm{G}}{\rm{V}}\mathop{{\rm{R}}}\limits_{\_}{\rm{P}}\mathop{D}\limits_{\_}{\rm{Q}}{\rm{V}}{\rm{C}}{\rm{S}}{\rm{Q}}{\rm{A}}{\rm{G}}{\rm{L}}{\rm{C}}\mathop{{\bf{F}}}\limits_{\_}{\rm{V}}\,\,(6990\,{{\rm{M}}}^{-1}{{\rm{c}}{\rm{m}}}^{-1})\\ {\rm{X}}:\quad \quad \quad \,\,\,\mathop{D}\limits_{\_}{\rm{G}}{\rm{A}}{\rm{Q}}{\rm{H}}{\rm{V}}{\rm{S}}{\rm{S}}{\rm{N}}{\rm{I}}\mathop{{\rm{K}}}\limits_{\_}{\rm{T}}{\rm{V}}{\rm{V}}\mathop{E{\rm{R}}E}\limits_{\_}{\rm{T}}\mathop{E}\limits_{\_}{\rm{G}}{\rm{S}}{\rm{S}}{\rm{V}}{\rm{G}}\,({\rm{n}}{\rm{o}}\,{\rm{a}}{\rm{b}}{\rm{s}}{\rm{o}}{\rm{r}}{\rm{p}}{\rm{t}}{\rm{i}}{\rm{v}}{\rm{i}}{\rm{t}}{\rm{y}})\\ {\rm{H}}3:\quad \quad \quad \mathop{E}\limits_{\_}{\rm{A}}{\rm{P}}{\rm{L}}{\rm{C}}{\rm{T}}{\rm{A}}{\rm{C}}\mathop{E}\limits_{\_}{\rm{M}}{\rm{A}}{\rm{V}}{\rm{V}}\mathop{{\bf{W}}}\limits_{\_}{\rm{M}}{\rm{Q}}{\rm{N}}{\rm{Q}}{\rm{L}}\mathop{{\rm{K}}}\limits_{\_}{\rm{Q}}\,(5500\,{{\rm{M}}}^{-1}{{\rm{c}}{\rm{m}}}^{-1})\\ {\rm{H}}3\,{\rm{E}}64{\rm{Q}}:\,\,\,{\rm{Q}}{\rm{A}}{\rm{P}}{\rm{L}}{\rm{C}}{\rm{T}}{\rm{A}}{\rm{C}}\mathop{E}\limits_{\_}{\rm{M}}{\rm{A}}{\rm{V}}{\rm{V}}\mathop{{\bf{W}}}\limits_{\_}{\rm{M}}{\rm{Q}}{\rm{N}}{\rm{Q}}{\rm{L}}\mathop{{\rm{K}}}\limits_{\_}{\rm{Q}}\,(5500\,{{\rm{M}}}^{-1}{{\rm{c}}{\rm{m}}}^{-1})\\ {\rm{H}}3\,{\rm{E}}72{\rm{Q}}:\,\,\mathop{E}\limits_{\_}{\rm{A}}{\rm{P}}{\rm{L}}{\rm{C}}{\rm{T}}{\rm{A}}{\rm{C}}{\rm{Q}}{\rm{M}}{\rm{A}}{\rm{V}}{\rm{V}}\mathop{{\bf{W}}}\limits_{\_}{\rm{M}}{\rm{Q}}{\rm{N}}{\rm{Q}}{\rm{L}}\mathop{{\rm{K}}}\limits_{\_}{\rm{Q}}\,(5500\,{{\rm{M}}}^{-1}{{\rm{c}}{\rm{m}}}^{-1})\\ {\rm{H}}3\,{\rm{K}}83{\rm{Q}}:\,\mathop{E}\limits_{\_}{\rm{A}}{\rm{P}}{\rm{L}}{\rm{C}}{\rm{T}}{\rm{A}}{\rm{C}}\mathop{E}\limits_{\_}{\rm{M}}{\rm{A}}{\rm{V}}{\rm{V}}\mathop{{\bf{W}}}\limits_{\_}{\rm{M}}{\rm{Q}}{\rm{N}}{\rm{Q}}{\rm{L}}{\rm{Q}}{\rm{Q}}\,(5500\,{{\rm{M}}}^{-1}{{\rm{c}}{\rm{m}}}^{-1})\\ {\rm{H}}4:\quad \quad \,\,\,\,\,\mathop{E}\limits_{\_}{\rm{G}}{\rm{T}}\mathop{K{\rm{E}}K}\limits_{\_}{\rm{V}}{\rm{L}}\mathop{E}\limits_{\_}\mathop{{\bf{Y}}}\limits_{\_}{\rm{V}}{\rm{N}}{\rm{Q}}{\rm{L}}{\rm{C}}{\rm{E}}{\rm{K}}{\rm{I}}{\rm{P}}\,(1490\,{{\rm{M}}}^{-1}{{\rm{c}}{\rm{m}}}^{-1})\end{array}$$

All peptides (with the exception of H1) were solubilised to a final concentration of 0.2 mg/mL by addition of in 20 mM sodium phosphate pH 7.2/10 mM DTT and gentle rocking for 135 min at ambient temperature followed by centrifugation at 10,000 × g for 5 min. Due to low solubility, H1 was prepared alternatively by solubilising in 6 mM NaOH followed by neutralisation with 0.05 volumes of 1 M sodium phosphate pH 7.2. The maximum concentration attained for H1 stock solution was 0.05 g/mL (18.5 µM). DTT was then added to 10 mM final concentration. The concentrations of H1, H1H2, H3, H3 mutants, and H4 were verified by A_280_. The concentrations of H2 and X, having no absorptivity, relied upon weight of de-salted, lyophilised material. This was deemed sufficient since H2 and X were both inactive and their concentrations were not otherwise needed for quantitative analyses. All peptides were stored at −30 °C after flash freezing in liquid nitrogen.

### Statistical Analyses

Statistical significance of differences within and between data sets were calculated with Kruskal-Wallis 1-way ANOVA and Dunn’s Multiple Comparison Test using GraphPad Prism 6.07.

### Data Availability

The datasets generated during and/or analysed during the current study are available from the corresponding author on reasonable request.

## Electronic supplementary material

Supplementary Information
