# Supplementary material for: Protein Structure Insights into the Bilayer Interactions of the Saposin-Like Domain of Solanum tuberosum Aspartic Protease
Source: Sci Rep. 2017 Dec 5;7:16911. doi: 10.1038/s41598-017-16734-2 (PMC5717070; doi:10.1038/s41598-017-16734-2)
Supplement: Supplementary file 1 — Supplementary Information [file 41598_2017_16734_MOESM1_ESM.pdf]

Protein Structure Insights into the Bilayer Interactions of the Saposin-Like Domain of *Solanum tuberosum* Aspartic Protease

[1]Brian C. Bryksa and [2*]Rickey Y. Yada

[1] Ontario Agricultural College, University of Guelph, Guelph, Ontario, Canada N1G 2W1
[2] Faculty of Land and Food Systems, University of British Columbia, Vancouver, British Columbia, Canada V6T 1Z4; Phone: +1-604–822–1219; E-mail: rickey.yada@ubc.ca
*To whom correspondence should be addressed

Supplementary Table S1: Proteins in the RCSB PDB databank that contain the [N/Q]-[N/Q]-[N/Q]-[A/L/I/V]-[R/K]-[N/Q] sequence motif.

PDB ID	PDB Macromolecule
3RFI	Saposin-like domain of plant aspartic protease from *Solanum tuberosum*
3HSI	Phosphatidylserine synthase *Haemophilus influenzae* Rd KW20
3KN1	Golgi phosphoprotein 3 N-term truncation variant
3U0C	N-terminal region of type III secretion first translocator Invasin IpaB
1XS5	Membrane lipoprotein TpN32 from *Treponema pallidum*
5AL6	Central coiled-coil domain of *Drosophila melanogaster* anastral spindle 2
1BWM	Single-chain T-cell receptor D10 from major histocompatibility complex class II-restricted
4U49	Pectate Lyase Pel3 from *Pectobacterium carotovorum*
4ULV	Cytochrome C prime from *Shewanella frigidimarina*
4QGO	DNA-entry nuclease (NucA) from *Streptococcus agalactiae*
4Q98	Fimbrilin - Major fimbrial subunit protein from *Porphyromonas gingivalis*
4LIK	Catalytic subunit of human DNA primase
4HOW	Isomaltulose synthase from *Erwinia rhapontici* NX5
4IGL	RHS-repeat containing B,C component of the secreted ABC toxin complex (*Yersinia entomophaga*)
4I5S	Sensor histidine kinase - Putative histidine kinase CovS; VicK-like protein
2LYI	Repetitive domain (RP) of aciniform spidroin 1 from *Nephila antipodiana*
3W14	Insulin receptor domains - L1,CR,L2 ,FNIII-1 alphact peptide
4DR0	*Bacillus subtilis* dimanganese(II) Ribonucleoside-diphosphate reductase subunit beta
3MVP	TetR/AcrR transcriptional regulator from *Streptococcus mutans*
3A09	*Sphingomonas* sp. A1 alginate-binding protein AlgQ1
2WDQ	*E. coli* succinate dehydrogenase hydrophobic membrane anchor subunit
3GBE	Isomaltulose synthase SmuA from *Protaminobacter rubrum*
3F3S	Human lambda-crystallin CRYL1
2EJ9	Riboflavin biosynthesis protein - Biotin protein ligase from *Methanococcus jannaschii*
2G6V	RibD from *E. coli*
2HR7	Insulin receptor domains 1-3
1YCK	Human peptidoglycan recognition protein PGRP-S
4ZXB	Human insulin receptor ectodomain - heavy chain, light chain

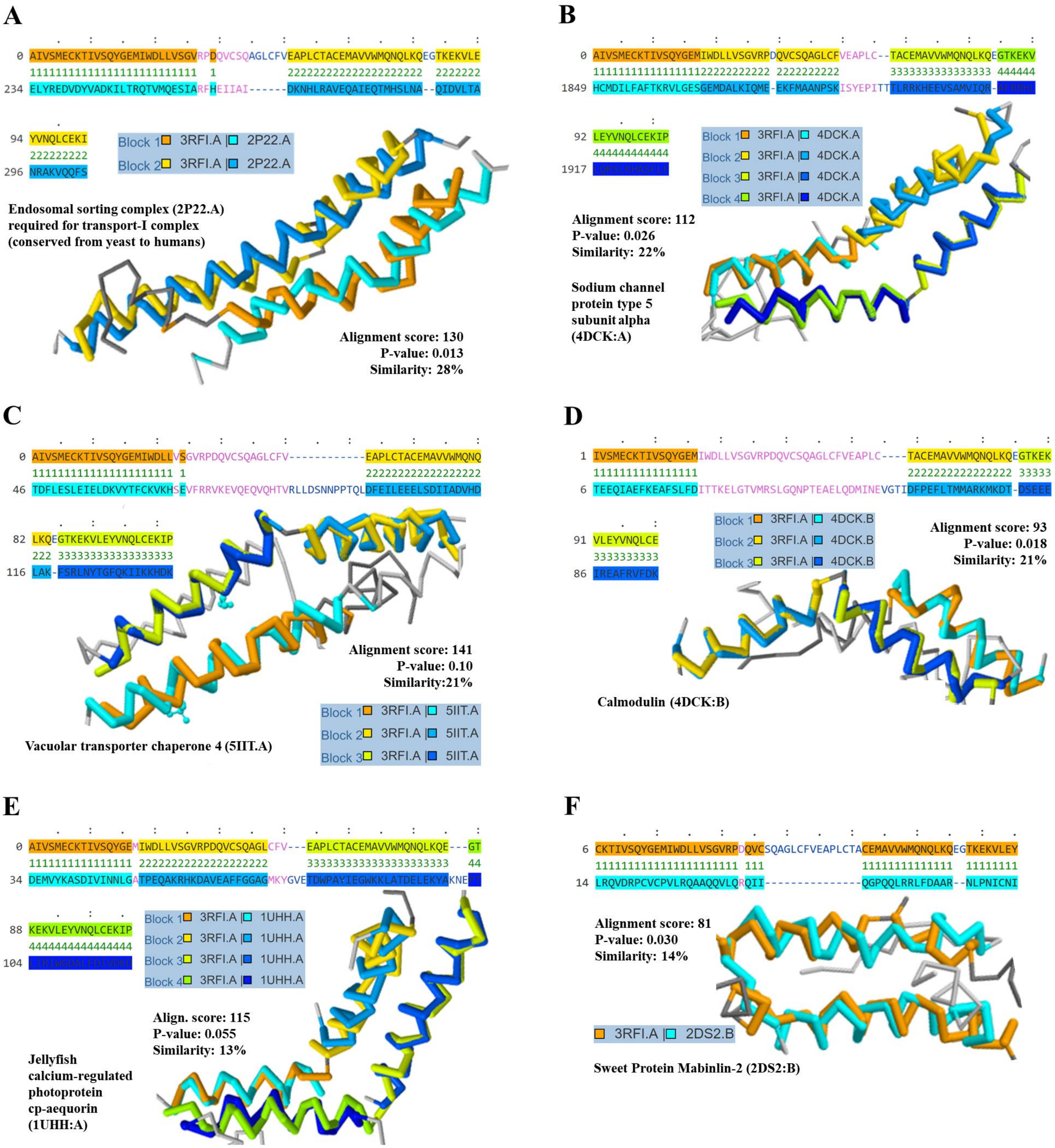

Supplementary Figure S1: Protein domain structure alignments. A protein domain structure alignment search of the RCSB PDB database using the Protein Structure Comparison Tool v.4.2.0 running the FATCAT algorithm yielded matches to PSI (3RFI) including diverse surface active/membrane interacting proteins. The alignments presented are selections based upon a combination of high alignment score, low P-value and/or high sequence similarity: (**A**) endosomal sorting complex; (**B**) sodium channel protein type 5 subunit α; (**C**) vacuolar transport chaperone 4; (**D**) calmodulin; (**E**) jellyfish calcium-regulated photoprotein; and (**F**) sweet protein mabinlin-2.

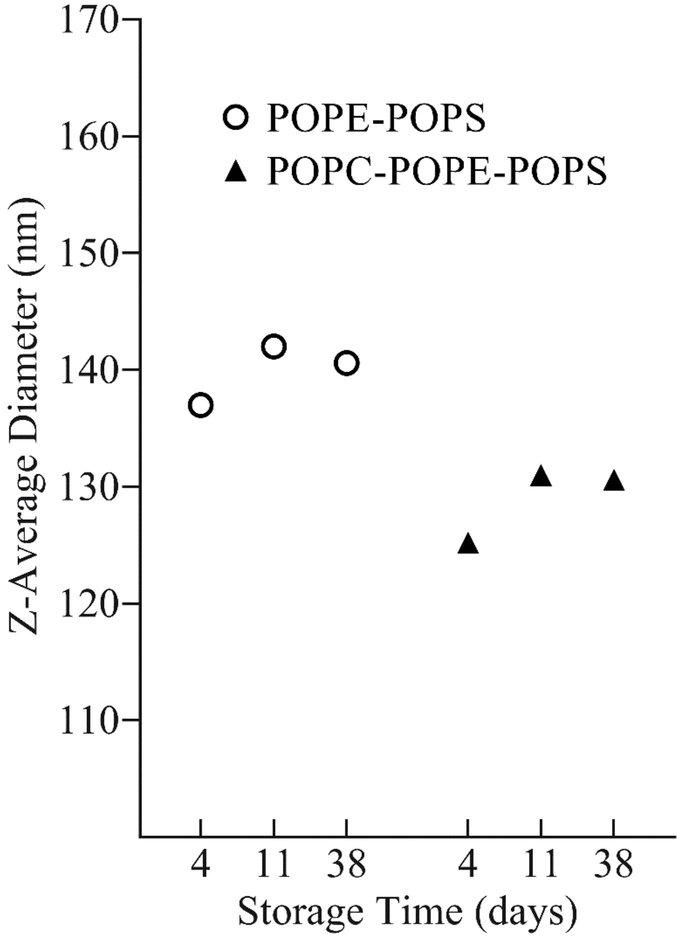

Supplementary Figure S2: Vesicle size monitored upon storage of LUV (1000 µM total phospholipid) stocks in the dark at ambient temperature for 4, 11, and 38 days.
